# Reduction of Financial Health Incentives and Changes in Physical Activity

**DOI:** 10.1001/jamanetworkopen.2023.42663

**Published:** 2023-11-08

**Authors:** Sean Spilsbury, Piotr Wilk, Carolyn Taylor, Harry Prapavessis, Marc Mitchell

**Affiliations:** 1 School of Kinesiology, Faculty of Health Sciences, Western University, London, Ontario, Canada; 2 Schulich School of Medicine, Department of Epidemiology and Biostatistics, Western University, London, Ontario, Canada; 3 Department of Statistics, Faculty of Sciences, University of British Columbia, Vancouver, British Columbia, Canada

## Abstract

**Importance:**

Governments and others continue to use financial incentives to influence citizen health behaviors like physical activity. However, when delivered on a population scale they can be prohibitively costly, suggesting more sustainable models are needed.

**Objectives:**

To evaluate the association of incomplete financial incentive withdrawal (“schedule thinning”) with physical activity after more than a year of incentive intervention and to explore whether participant characteristics (eg, app engagement and physical activity) are associated with withdrawal outcomes.

**Design, Setting, and Participants:**

This case-control study with a pre-post quasi-experimental design included users of a government-funded health app focused on financial incentives. Eligible participants were residents in 3 Canadian provinces over 25 weeks in 2018 and 2019. Data were analyzed from July 2021 to December 2022.

**Exposure:**

Due to fiscal constraints, financial incentives for daily physical activity goals were withdrawn in Ontario in December 2018 (case)—representing a 90% reduction in incentive earnings—but not in British Columbia or Newfoundland and Labrador (controls).

**Main Outcome and Measures:**

The primary outcome was objectively assessed weekly mean daily step count. Linear regression models were used to compare pre-post changes in daily step counts between provinces (a difference-in-differences approach). Separate models were developed to examine factors associated with changes in daily step count (eg, app engagement and physical activity). Clinically meaningful initial effect sizes were previously reported (approximately 900 steps/d overall and 1800 steps/d among the physically inactive).

**Results:**

In total there were 584 760 study participants (mean [SD] age, 34.3 [15.5] years; 220 388 women [63.5%]), including 438 731 from Ontario, 124 101 from British Columbia, and 21 928 from Newfoundland and Labrador. Significant physical activity declines were observed when comparing pre-post changes in Ontario to British Columbia (−198 steps/d; 95% CI, −224 to −172 steps/d) and Newfoundland and Labrador (−274 steps/d; 95% CI, −323 to −225 steps/d). The decrease was most pronounced for highly engaged Ontario users (−328 steps/d; 95% CI, −343 to −313 steps/d). Among physically inactive Ontario users, physical activity did not decline following withdrawal (107 steps/d; 95% CI, 90 to 124 steps/d).

**Conclusions and Relevance:**

In this case-control study of incomplete financial incentive withdrawal, statistically significant daily step count reductions were observed in Ontario; however, these declines were modest and not clinically meaningful. Amidst substantial program savings, the physical activity reductions observed here may be acceptable to decision-makers working within finite budgets.

## Key Points

**Question:**

Can financial health incentive programs be mostly scaled back without untoward effects on health behaviors?

**Findings:**

In this case-control study using a large natural experiment design with 584 760 participants, financial health incentive withdrawal after more than a year of incentive intervention led to statistically significant, but modest and not clinically meaningful, physical activity declines.

**Meaning:**

These results suggest that physical activity, once established, may be maintained with less frequent and less costly financial health incentive reinforcement.

## Teaser

This case-control study of Canadian users of a phone app focused on physical fitness and health examines the association of partial withdrawal of financial incentives with physical activity.

## Introduction

Habitual physical activity reduces the risk of over 100 chronic conditions and yet widespread inactivity persists.^1^ Digital tools (eg, mHealth apps) may help promote physical activity but are characterized by low engagement and small or no effects.^2^ Financial incentives, such as paying people to be active, have emerged as one possible solution.^3,4^ When delivered on a population scale, though, they can be prohibitively costly—a critical limitation. Calls from governments, corporations, and scholars continue to ring out for sustainable financial incentive models.^5^ One example of such a model drawn from a list of published financial incentive design features^6^ includes limited time only reinforcement schedules, where financial incentives are offered for a period (ideally until habit formation) and then are either completely or incompletely withdrawn (referred to as “schedule thinning”). Unfortunately, few have examined the impact of schedule thinning on physical activity even though it is theorized to protect established behaviors.^7,8^ A commercial mHealth app that was available for download in Canada between 2016 and 2019^9^ presents a unique opportunity to explore the effects of incomplete financial incentive withdrawal on physical activity in a large-scale public health context. In brief, an abrupt program change in 2018 divided the app user base into exposed (ie, incomplete financial incentive withdrawal) and unexposed (ie, no financial incentive withdrawal) groups. The overarching objective of this study was to use this naturally occurring variation in exposure to explore the association of incomplete withdrawal with changes in physical activity.

## Methods

This study follows the Strengthening the Reporting of Observational Studies in Epidemiology (STROBE) reporting guidelines for case-control studies. Ethical approval for this study was provided by the Western University Human Research Ethics Board. Informed consent was not required as this research relies exclusively on the secondary use of nonidentifiable data.

### Setting

Between 2016 and 2019, Carrot was available for download in the Canadian provinces of British Columbia (Spring 2016 launch), Newfoundland and Labrador (Spring 2016 launch), and Ontario (Winter 2017 launch). A list of behavior change techniques (eg, goal setting, self-monitoring)^10^ including features of the app’s main intervention component, financial incentives,^6^ is provided in eTables 1 and 2 in Supplement 1. The app’s cornerstone feature, Steps, offered users very small provincially funded financial incentives worth $0.04 CAD per day for completing personalized and adaptive daily step count goals (ie, bimonthly personalized goals calculated using the median daily step count from the previous 30 days plus 10%). In addition to these daily financial incentives, harder-to-earn team-based physical activity rewards worth $0.40 CAD per week were also available (ie, users earn by collectively completing 10 or more personalized daily step goals in 7 days with a partner) (Steps feature overview in eAppendix 1 in Supplement 1). The app leveraged principles from behavioral economics, an offshoot of traditional economics complemented by insights from psychology,^11^ to boost incentive effects and keep incentive costs low (eTable 3 in Supplement 1). Notably, while the financial incentives used were very small, they were provided instantaneously using smartphone technology for individual- and team-based successes in the form of loyalty points (eg, for cinema tickets, gas) that people tend to overvalue. Indeed, recent evidence suggests that reward size may be less important than other financial incentive intervention design features (eg, timing or form).^12^

A longitudinal (ie, 12-month) quasi-experimental investigation of the multicomponent app found that, among users with complete data, physical activity increased by 885 steps/d (95% CI, 825-944 steps/d; Cohen *f^2^* = 0.056).^13^ The increase was 2-fold among physically inactive users (1821 steps/d; 95% CI, 1739-1902 steps/d; Cohen *f^2^* = 0.314). While there is limited evidence regarding the minimum number of daily steps needed for clinically meaningful change,^14^ a systematic review of large prospective studies by Hall et al^15^ found that 1000 is consistently associated with lower morbidity (eg, cardiovascular disease incidence) and mortality (eg, all-cause) risk. A 2022 meta-analysis^16^ also suggests a lower threshold may exist for those with lower step volumes (eg, below 5000 steps/d). Among those living with chronic disease, for example, there is evidence that smaller increments (eg, 750 steps/d) could have clinically meaningful health benefits.^17,18^ Due to fiscal constraints, in December 2018, financial incentives for personalized daily physical activity goals were withdrawn for app users in Ontario (case)—representing a 90% reduction in financial incentive for physical activity earnings—but not in British Columbia or Newfoundland and Labrador (controls). In all 3 provinces financial incentives for team-based weekly physical activity goals persisted representing the remaining 10%. While this incomplete financial incentive withdrawal was driven by economic necessity rather than by theory or hypothesis testing, it did introduce the program variance needed for a large-scale natural experiment.

### Study Design

**Figure 1.**
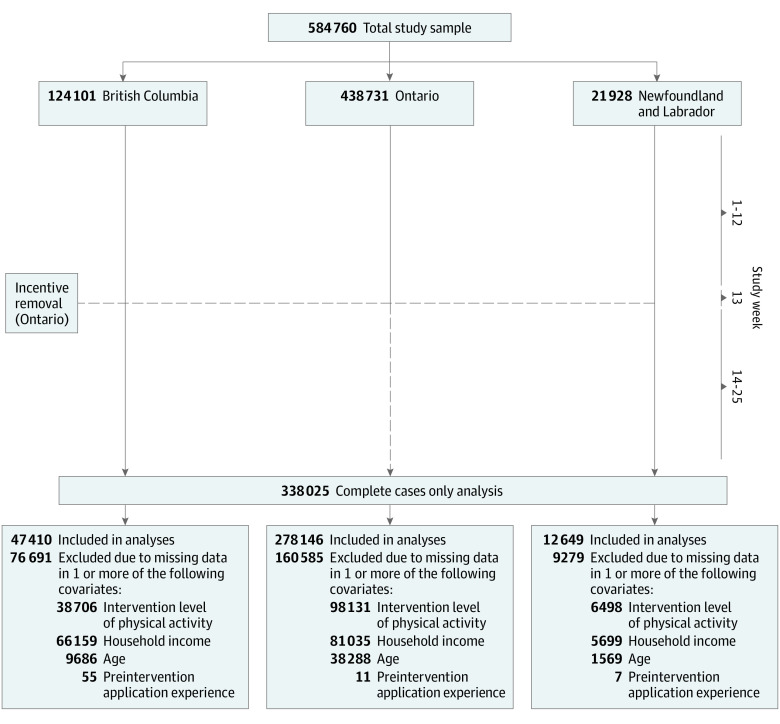
Study Flowchart

**Figure 2.**
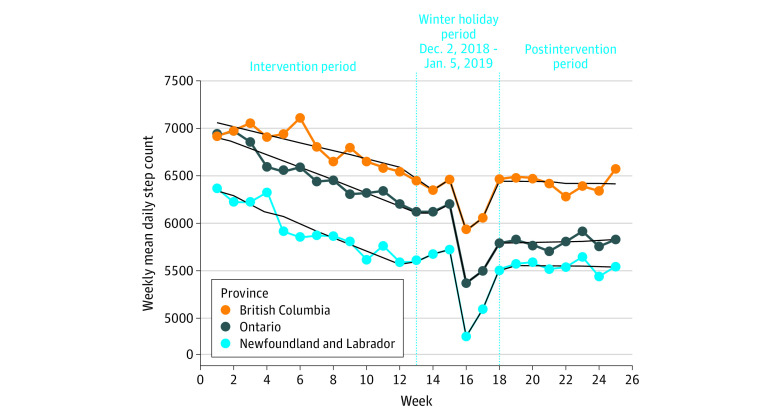
Mean Weekly Daily Step Count by Week and Intervention Period, Complete Cases Sample

To examine this naturally occurring experiment a 25-week quasi-experimental case-control study was adopted using a retrospective pre-post design with 2 nonequivalent control groups (Figure 1). The intervention period was defined as the 12 weeks before incomplete financial incentive withdrawal in Ontario (study weeks 1 to 12; September 9 to December 2, 2018). An email notification was sent to app users in Ontario at the beginning of study week 13 (December 3, 2018) announcing that incomplete withdrawal was to take effect later that week (December 8, 2018). Since the Canadian winter holiday season (December 9, 2018, to January 5, 2019; study weeks 14 to 17) could have extraneously influenced daily step count during the evaluation period, study weeks 13 to 17 were treated as a pseudo-washout period to allow physical activity behaviors to stabilize prior to examining the impact of incomplete financial incentive withdrawal (Figure 2). The postintervention period was therefore defined as the final 8 weeks of the study (study weeks 18 to 25; January 6 to March 2, 2019). The primary study objective was to examine the impact of incomplete financial incentive withdrawal on physical activity in Ontario after more than a year of financial incentive intervention compared with British Columbia and Newfoundland and Labrador, where financial incentive availability did not change. The secondary objective was to explore whether participant characteristics (eg, app engagement and physical activity) were associated with withdrawal effects.

### Outcome Measure

The outcome measure was individual-level weekly mean daily step count measured by built-in smartphone accelerometers (study weeks 1 through 25). Validation studies have shown that the step counting feature in iPhone and Android smartphones are accurate when compared with criterion standards of measurement.^19^ Only days with step counts between 1000 and 40 000 (referred to as valid days) were used to calculate weekly mean daily step count as these were considered reasonable (not outliers).^20^

### Covariates

Previous financial incentives for physical activity studies informed covariate selection a priori given their potential association with the outcomes of incomplete financial incentive removal on physical activity, including (1) intervention period physical activity level (eg, physically inactive, equaling less than 5000 steps/d), (2) intervention period app engagement (eg, low, equaling less than 5 weeks with 1 or more app opens per week), (3) total app experience (eg, low, equaling less than 6 months), and (4) sociodemographics (eTable 4 in Supplement 1). Notably, app engagement was defined using frequency of app use only (vs depth of use [ie, time on app]), a common objective measure of app engagement.^21,22^ Since Steps was the app’s cornerstone feature, ideally used often (ie, at least weekly) but not necessarily for very long (ie, less than 2 minutes), the operational definition of app engagement in this study was number of weeks with at least 1 app open.

### Statistical Analyses

Two analytical approaches were used to account for missing data and to test the sensitivity of assumptions with the analytical sample: (1) the complete cases approach included participants with weekly mean daily step count data in the intervention and postintervention periods as well as complete measures for each covariate (338 025 users); (2) the multiple imputation approach using 5 imputed data sets, created by imputing missing data of effected variables through sampling from the estimated distribution of observed data, included all participants (584 760 users). Since the 2 analytic approaches yielded very similar results, analyses using the more conservative complete cases approach are presented as these participants were more engaged with the app and thus more likely to respond to incomplete financial incentive withdrawal (ie, experience a physical activity drop). A χ^2^ test of independence and independent-samples Kruskal-Wallis tests were conducted to identify baseline characteristic differences between complete cases and multiple imputation analytic samples, as well as users excluded from the complete cases sample.

Statistical analyses were completed using RStudio version 4.0.5 (RStudio). To address the primary study objective, a multiple linear regression model with a robust sandwich estimator of covariance was fit to weekly mean daily step count that included a 2-way interaction between a 25-level categorical variable for study week (1 to 25) and a 7-level categorical variable for period (intervention, washout period [week 13, week 14, …, week 17], and postintervention). This fit a straight line through study weeks 1 to 12 (intervention period) as well as through study weeks 18 to 25 (postintervention period) along with separate average estimates for each of study weeks 13 to 17. All covariates were included in the model as main effects to address selection bias at least partly. The association of incomplete financial incentive withdrawal with weekly mean daily step count was assessed by calculating the difference of the intervention intercept (study week 12, the intervention period end point) to the postintervention intercept (study week 21, the postintervention period midpoint) within each province. These differences were then compared between provinces (ie, a difference-in-differences approach).^23^ These 3 comparisons (ie, Ontario vs British Columbia, Ontario vs Newfoundland and Labrador, British Columbia vs Newfoundland and Labrador) were completed using SE estimates of the intervention to postintervention intercept difference for each province to calculate the unpooled SE and significance was assessed using a 2-tailed *z* test. The statistical significance of estimated postintervention period slopes was also assessed, with a threshold of significance of *P* < .05 in 2-sided tests. The multiple linear regression model used for the primary analysis is presented in eAppendix 2 in Supplement 1. To address the secondary study objective, another multiple linear regression model using the complete cases sample included a 3-way interaction between covariate level, study week, and period. Separate models were used to analyze the 3-way interaction for each covariate.

## Results

### Sample Characteristics

In total there were 584 760 study participants (mean [SD] age: 34.3 [15.5] years; 220 388 women [63.5%]). The complete cases sample consisted of 338 025 participants from Ontario (278 146 [75.0%]), British Columbia (47 410 [14.0%]), and Newfoundland and Labrador (12 469 [3.7%]), representing 57.8% of the total (ie, multiple imputation) sample (Table 1). Fewer British Columbia users were included in the complete cases sample (38.2%) than Ontario and Newfoundland and Labrador users (63.4% and 57.7%, respectively) because household income was available for a smaller proportion of British Columbia users (46.7% vs 81.5% in Ontario and 74.0% in Newfoundland and Labrador), one of the covariates identified a priori as a factor. As well, provincial total app experience, intervention period app engagement and physical activity data for the complete cases sample are shown in eTable 5 in Supplement 1. Characteristics of the multiple imputation sample (584 760 users) as well as of those excluded from the complete cases sample (246 735 users) are in eTables 6 through 8 in Supplement 1. Notably, participants excluded from the complete cases sample were less engaged with the app.

**Table 1.** Baseline Characteristics, Complete Cases Sample

Characteristic	Participants, No. (%) (N = 338 025)		
	Ontario (n = 278 146)	British Columbia (n = 47 410)	Newfoundland and Labrador (n = 12 469)
Age, mean (SD), y	33.9 (12.7)	36.5 (13.3)	35.8 (12.8)
Gender			
Female	179 744 (64.6)	31 684 (66.8)	8960 (71.9)
Male	94 365 (33.9)	14 784 (31.2)	3398 (27.2)
Other^a^	4037 (1.5)	942 (2.0)	111 (0.9)
Annual household income, CAD $^b^			
<20 000	25 896 (9.3)	3868 (8.2)	1083 (8.7)
20 000 to <40 000	34 575 (12.4)	5919 (12.5)	1474 (11.8)
40 000 to <60 000	42 182 (15.2)	7777 (16.4)	1725 (13.8)
60 000 to <80 000	35 961 (12.9)	6375 (13.5)	1494 (12.0)
80 000 to <100 000	29 271 (10.5)	5039 (10.6)	1374 (11.0)
100 000 to <150 000	31 579 (11.4)	5741 (12.1)	1705 (13.7)
≥150 000	23 030 (8.3)	3321 (7.0)	1120 (9.0)
Did not complete	3220 (1.2)	337 (0.7)	121 (1.0)
Do not know	11 172 (4.0)	1470 (3.1)	366 (2.9)
Rather not say	41 260 (14.8)	7563 (15.9)	2007 (16.1)
Loyalty rewards program^c^			
Aeroplan miles	41 084 (14.8)	8595 (18.1)	3320 (26.6)
Drop points	12 605 (4.5)	1615 (3.4)	598 (4.8)
More Rewards	401 (0.1)	4856 (10.3)	22 (0.2)
Petro-points	30 244 (10.9)	2989 (6.3)	87 (0.7)
RBC rewards	5724 (2.1)	1040 (2.2)	212 (1.7)
Scene+ points	188 088 (67.6)	28 315 (59.7)	8230 (66.0)
Baseline step count, mean (SD), steps/d^d^	5751 (3714)	5883 (3513)	5307 (3485)

Abbreviations: CAD, Canadian dollars; RBC, Royal Bank of Canada.

^a^ Identified gender was neither woman nor man.

^b^ Mean exchange rate in 2018: CAD $1.3449 = US $1.00.

^c^ Loyalty rewards programs explanation, by category: Aeroplan miles provides credit for air travel; Drop points, miscellaneous consumer goods; More Rewards, groceries; Petro-Points, gasoline; RBC rewards, bank credit; Scene+ points, cinema tickets.

^d^ Mean daily step count over a 7-day period prior to app intervention exposure.

### Primary and Sensitivity Analyses

The parallel downward trend in all 3 provinces from study weeks 1 to 13 reflected typical seasonal physical activity decline in Canada (moving from the tepid fall to the colder winter season) and not waning intervention effectiveness (Figure 2). Estimated intercept values dropped from intervention (study week 12; intervention period end point) to postintervention (study week 21; postintervention period midpoint) in all 3 provinces with the most pronounced decrease noted in Ontario (Ontario, −367 steps/d; 95% CI, −377 to −357 steps/d; British Columbia, −169 steps/d; 95% CI, −193 to −145 steps/d; Newfoundland and Labrador, −93 steps/d; −140 to −46 steps/d) (Table 2). In addition, the intervention to postintervention intercept difference was greatest when comparing Ontario with British Columbia (−198 steps/d; 95% CI, −224 to −172 steps/d) and Newfoundland and Labrador (−274 steps/d; 95% CI, −323 to −225 steps/d) (Table 3). Regarding postintervention weekly mean daily step count slopes, the rates of change were modest in terms of steps per day (eTable 9 in Supplement 1). Lastly, the results from sensitivity analyses (ie, multiple imputation) were generally consistent with the complete cases analysis (eAppendix 3, eTables 10-12 in Supplement 1).

**Table 2.** Estimated Weekly Mean Daily Step Count Intercepts (Within Province), Complete Cases Sample

Parameter	β̂ (SE) [95% CI]					
	Ontario	*P* value	British Columbia	*P* value	Newfoundland and Labrador	*P* value
Intercept						
Intervention (week 12)	6153 (3.05) [6147-6159]	NA	6535 (7.27) [6520-6549]	NA	5574 (14.02) [5547-5602]	NA
Postintervention (week 21)	5786 (4.34) [5777-5794]	NA	6366 (9.62) [6347-6385]	NA	5481 (19.75) [5442-5520]	NA
Difference^a^	367 (5.30) [357-377]	<.001	169 (12.10) [145, 193]	<.001	93 (24.20) [46-140]	<.001

Abbreviations: β̂, unstandardized regression coefficient; NA, not applicable.

^a^ Intervention to postintervention weekly mean daily step count difference.

**Table 3.** Difference in the Intervention to Postintervention Weekly Mean Daily Step Count Intercept Differences (Between Provinces), Complete Cases Sample

Parameter	β̂ (SE) [95% CI]					
	Ontario vs British Columbia	*P* value	Ontario vs Newfoundland and Labrador	*P* value	British Columbia vs Newfoundland and Labrador	*P* value
Difference-in- differences^a^	198 (13.21) [172-224]	<.001	274 (24.80) [225-323]	<.001	76 (27.13) [23-129]	<.001

Abbreviation: β̂, unstandardized regression coefficient.

^a^ Difference-in-differences describes the difference in the between-province intervention to postintervention weekly mean daily step count intercept differences.

### Secondary Analysis

When comparing estimated intervention and postintervention weekly mean daily step count intercepts and slopes for covariates from the complete cases sample, the estimated intercept decrease from intervention to postintervention was more pronounced among highly engaged and physically active users in Ontario (high engagement, −328 steps/d [95% CI, −343 to −313 steps/d]; low engagement, −211 steps/d [95% CI, −255 to −167 steps/d]; physically active, −232 steps/d [95% CI, −247 to −217 steps/d]; physically inactive, 107 steps/d [90 to 124 steps/d]) (eTables 13-22 in Supplement 1). Physically inactive users were the only group to exhibit an increase in estimated intercept from intervention to postintervention in Ontario (107 steps/d; 95% CI, 90 to 124 steps/d). Similarly, physically inactive British Columbia (234 steps/d; 95% CI, 192 to 276 steps/d) and Newfoundland and Labrador (187 steps/d; 95% CI, 121 to 253 steps/d) users also exhibited increases. Total app experience, age, and gender were not associated with the estimated intercept decrease from intervention to post-intervention in Ontario. Overall, data generally showed covariates to only modestly affect postintervention estimated slopes (eTables 13-22 in Supplement 1).

## Discussion

Financial incentives may have a role to play in increasing physical activity. However, when delivered on a population scale, they can be prohibitively costly. Sustainable financial incentive models are needed. In the absence of a robust randomized clinical trial (RCT) evidence base, rigorous evaluations of naturally occurring financial incentive program changes (eg, incomplete financial incentive withdrawal) may shed light on this timely public health question. This quasi-experimental study revealed modest physical activity reductions following incomplete financial incentive withdrawal—approximately 100 to 300 steps/d depending on the analytic approach (ie, complete cases vs multiple imputation) and the subgroup analyzed (eg, highly engaged). These reductions are not clinically meaningful.^15^ In addition, these reductions represent a small proportion of the initial effect calculated by Mitchell et al^13^ after 1 year of exposure to the app (ie, approximately 900 steps/d in general and 1800 steps/d for physically inactive users specifically). After slashing financial incentive costs by 90%, then, daily step count dropped by only 10% to 25%, albeit over a relatively short postintervention evaluation period (ie, 8 weeks). One reason for these modest declines might be that rewards for daily physical activity achievements were provided for over a year before withdrawal began, likely enough time for habit formation.^24^ These results suggest that physical activity, once established, may be maintained with less frequent and less costly financial incentive reinforcement. While study data were collected 5 years ago (before the World Health Organization declared COVID-19 a pandemic),^25^ the findings are still relevant as governments and corporations continue to use financial incentives to influence citizen health behaviors.^26,27,28,29,30^

Only 2 RCTs to our knowledge have investigated the impact of incomplete financial incentive withdrawal on physical activity in adults.^31,32^ The results presented here generally align with findings from Pope et al^31^ and Andrade et al.^32^ In each, behavioral economics–informed reinforcement schedule thinning (ie, incomplete financial incentive withdrawal) appeared to sustain physical activity to a degree. In Pope et al,^31^ for example, gym visits fell by 38% following a 65% reduction in financial incentives. The very limited RCT evidence-base, then, suggests physical activity maintenance following incomplete financial incentive withdrawal may be possible (at least for a few weeks) but is probably not automatic. Incorporating insights from behavioral economics in financial incentive design, for example, as well as from other behavior change theories may increase the likelihood of behavior maintenance following financial incentive removal. Self-determination theory, for instance, is a global theory of human motivation made up of 4 mini-theories that focuses on the extent to which behaviors are controlled by external agents or contingencies (eg, financial incentives). Cognitive evaluation theory, a self-determination theory mini-theory defining social and environmental factors that promote more internalized motivation, suggests that providing external rewards in ways that promote the satisfaction of individuals’ basic psychological needs of (1) competence (ie, experiencing mastery), (2) autonomy (ie, sense of control over behavior), and (3) social relatedness (ie, feeling socially connected to others) may nurture intrinsic motivation and increase the potential for sustained change.^33^ Another reason why physical activity improvements may have been mostly maintained following incomplete financial incentive withdrawal in this study, therefore, may be because the app rewarded (1) the achievement of realistic and adaptive behavioral goals (ie, to increase competence) with (2) modest loyalty point incentives for daily-then-weekly achievements (ie, less controlling, autonomy promoting) for (3) small group successes (ie, weekly rewards were contingent on team success). This may have been particularly true for physically inactive app users—who have lower levels of perceived competence to begin with, and for whom physical activity did not drop following financial incentive withdrawal. Physically inactive users may have also been more apt to increase their physical activity as part of New Year resolutions.^34^

### Limitations

Several limitations must be considered when interpreting the results of this study. First, the impact of financial incentives within the multicomponent app could not be isolated in the first place given the public health programming nature of the intervention. However, our previous work suggests app engagement and effectiveness was driven largely by the incentives.^35,36^ Previous research also suggests that adding modest financial incentives to similar multicomponent mHealth interventions boosts physical activity by approximately 700 steps/d.^3,4^ For example, financial incentives accounted for 700 out of the 1050 daily step count increase observed in a 12-month RCT by Finkelstein et al.^37^ Another study limitation is that while external validity is prioritized within this quasi-experimental design (eg, focusing on the sustainability of a real world mHealth intervention), selection and history bias may threaten internal validity. To partly address selection bias with both complete cases and multiple imputation analyses, baseline (eg, income) and intervention period (eg, app experience) covariates were balanced between provinces with regression adjustment.^23,38^ As well, complete cases and multiple imputation analytic approaches yielded similar results mitigating concerns that selection bias threatens inferences of effect.^23,38^ History bias, where events unrelated to the intervention occur during the intervention period (eg, inclement weather) and influence the outcome (eg, physical activity), may also limit internal validity. But step count data at multiple intervention and postintervention time points (eg, the stable physical activity levels across provinces postintervention) show results that are closer to an interrupted time series design, providing support for our interpretations (Figure 2).^23,38^ Third, the 12-week postintervention period may not have been long enough to fully capture the impact of incomplete financial incentive withdrawal in Ontario. However, most RCTs monitoring physical activity into postincentive periods have observed declines to baseline levels within this timeframe,^37^ suggesting deleterious effects are observed right away.

## Conclusions

In this case-control study evaluating the impact of incomplete financial incentive withdrawal (commonly referred to as “schedule thinning”) on physical activity, withdrawal after more than a year of incentive intervention led to statistically significant, but modest and nonclinically meaningful, daily step count declines. The magnitude of these declines may be acceptable to decision makers working within finite budgets.

## Notes

Supplement 1. **eTable 1.** Behaviour Change Techniques (BCTs) **eTable 2.** Financial Incentive Design Features and Attributes **eAppendix 1.** Carrot Rewards mHealth App “Steps” Feature Overview **eTable 3.** Behavioral Economics Principles (ie, Decision Biases) Used in Financial Incentive Interventions **eTable 4.** Covariate Level Categories and Criteria **eAppendix 2.** Multiple Linear Regression Model, Primary Analysis **eTable 5.** Intervention Period Behaviours, Complete Cases (CC) Sample **eTable 6.** Baseline Characteristics, Multiple Imputation Sample **eTable 7.** Baseline Characteristics of Users Excluded From the Complete Cases Sample **eTable 8.** Intervention Period Behaviours of Users Excluded From the Complete Cases Sample **eTable 9.** Estimated Weekly Mean Daily Step Count Slopes (Within Provinces), Complete Cases (CC) Sample **eAppendix 3.** Sensitivity Analysis Results Summary **eTable 10.** Estimated Weekly Mean Daily Step Count Intercepts and Slopes (Within Provinces), Multiple Imputation Sample **eTable 11.** Estimated Weekly Mean Daily Step Count Intercepts and Slopes (Between Provinces), Multiple Imputation Sample **eTable 12.** Difference of the Intervention to Postintervention Weekly Mean Daily Step Count Change (Between Province), Multiple Imputation Sample **eTable 13.** Estimated Weekly Mean Daily Step Count Intercepts and Slopes by Application Engagement (Within Provinces), Complete Cases Sample **eTable 14.** Estimated Weekly Mean Daily Step Count Intercepts and Slopes by Application Engagement (Between Provinces), Complete Cases Sample **eTable 15.** Estimated Weekly Mean Daily Step Count Intercepts and Slopes by Intervention Level of Physical Activity (Within Provinces), Complete Cases Sample **eTable 16.** Estimated Weekly Mean Daily Step Count Intercepts and Slopes by Preintervention Level of Physical Activity (Between Provinces), Complete Cases Sample **eTable 17.** Estimated Weekly Mean Daily Step Count Intercepts and Slopes by Application Experience (Within Provinces), Complete Cases Sample **eTable 18.** Estimated Weekly Mean Daily Step Count Intercepts and Slopes by Application Experience (Between Provinces), Complete Cases Sample **eTable 19.** Estimated Weekly Mean Daily Step Count Intercepts and Slopes by Age (Within Provinces), Complete Cases Sample **eTable 20.** Estimated Weekly Mean Daily Step Count Intercepts and Slopes by Age (Between Provinces), Complete Cases Sample **eTable 21.** Estimated Weekly Mean Daily Step Count Intercepts and Slopes by Gender (Within Provinces), Complete Cases Sample **eTable 22.** Estimated Weekly Mean Daily Step Count Intercepts and Slopes by Gender (Between Provinces), Complete Cases Sample **eAppendix 4.** Code for Primary Data Analysis

Supplement 2.
Data Sharing Statement
