# Supplementary material for: Reduction of Financial Health Incentives and Changes in Physical Activity
Source: JAMA Netw Open. 2023 Nov 8;6(11):e2342663. doi: 10.1001/jamanetworkopen.2023.42663 (PMC10632955; doi:10.1001/jamanetworkopen.2023.42663)
Supplement: Supplement 1. — eTable 1. Behaviour Change Techniques (BCTs) eTable 2. Financial Incentive Design Features and Attributes eAppendix 1. Carrot Rewards mHealth App “Steps” Feature Overview eTable 3. Behavioral Economics Principles (ie, Decision Biases) Used in Financial Incentive Interventions eTable 4. Covariate Level Categories and Criteria eAppendix 2. Multiple Linear Regression Model, Primary Analysis eTable 5. Intervention Period Behaviours, Complete Cases (CC) Sample eTable 6. Baseline Characteristics, Multiple Imputation Sample eTable 7. Baseline Characteristics of Users Excluded From the Complete Cases Sample eTable 8. Intervention Period Behaviours of Users Excluded From the Complete Cases Sample eTable 9. Estimated Weekly Mean Daily Step Count Slopes (Within Provinces), Complete Cases (CC) Sample eAppendix 3. Sensitivity Analysis Results Summary eTable 10. Estimated Weekly Mean Daily Step Count Intercepts and Slopes (Within Provinces), Multiple Imputation Sample eTable 11. Estimated Weekly Mean Daily Step Count Intercepts and Slopes (Between Provinces), Multiple Imputation Sample eTable 12. Difference of the Intervention to Postintervention Weekly Mean Daily Step Count Change (Between Province), Multiple Imputation Sample eTable 13. Estimated Weekly Mean Daily Step Count Intercepts and Slopes by Application Engagement (Within Provinces), Complete Cases Sample eTable 14. Estimated Weekly Mean Daily Step Count Intercepts and Slopes by Application Engagement (Between Provinces), Complete Cases Sample eTable 15. Estimated Weekly Mean Daily Step Count Intercepts and Slopes by Intervention Level of Physical Activity (Within Provinces), Complete Cases Sample eTable 16. Estimated Weekly Mean Daily Step Count Intercepts and Slopes by Preintervention Level of Physical Activity (Between Provinces), Complete Cases Sample eTable 17. Estimated Weekly Mean Daily Step Count Intercepts and Slopes by Application Experience (Within Provinces), Complete Cases Sample eTable 18. Estimated Weekly [file jamanetwopen-e2342663-s001.pdf]

Supplemental Online Content

Spilsbury S, Wilk P, Taylor C, Prapavessis H, Mitchell M. Examining more sustainable financial health incentives in Canada. *JAMA Netw Open.* 2023;6(11):e2342663. doi:10.1001/jamanetworkopen.2023.42663

eTable 1. Behaviour Change Techniques (BCTs)

eTable 2. Financial Incentive Design Features and Attributes

eAppendix 1. Carrot Rewards mHealth App "Steps" Feature Overview

eTable 3. Behavioral Economics Principles (ie, Decision Biases) Used in Financial Incentive Interventions

eTable 4. Covariate Level Categories and Criteria

eAppendix 2. Multiple Linear Regression Model, Primary Analysis

eTable 5. Intervention Period Behaviours, Complete Cases (CC) Sample

eTable 6. Baseline Characteristics, Multiple Imputation Sample

eTable 7. Baseline Characteristics of Users Excluded From the Complete Cases Sample

eTable 8. Intervention Period Behaviours of Users Excluded From the Complete Cases Sample

eTable 9. Estimated Weekly Mean Daily Step Count Slopes (Within Provinces), Complete Cases (CC) Sample

eAppendix 3. Sensitivity Analysis Results Summary

eTable 10. Estimated Weekly Mean Daily Step Count Intercepts and Slopes (Within Provinces), Multiple Imputation Sample

eTable 11. Estimated Weekly Mean Daily Step Count Intercepts and Slopes (Between Provinces), Multiple Imputation Sample

eTable 12. Difference of the Intervention to Postintervention Weekly Mean Daily Step Count Change (Between Province), Multiple Imputation Sample

eTable 13. Estimated Weekly Mean Daily Step Count Intercepts and Slopes by Application Engagement (Within Provinces), Complete Cases Sample

eTable 14. Estimated Weekly Mean Daily Step Count Intercepts and Slopes by Application Engagement (Between Provinces), Complete Cases Sample

© 2023 Spilsbury S et al. *JAMA Network Open.*

eTable 15. Estimated Weekly Mean Daily Step Count Intercepts and Slopes by Intervention Level of Physical Activity (Within Provinces), Complete Cases Sample

eTable 16. Estimated Weekly Mean Daily Step Count Intercepts and Slopes by Preintervention Level of Physical Activity (Between Provinces), Complete Cases Sample

eTable 17. Estimated Weekly Mean Daily Step Count Intercepts and Slopes by Application Experience (Within Provinces), Complete Cases Sample

eTable 18. Estimated Weekly Mean Daily Step Count Intercepts and Slopes by Application Experience (Between Provinces), Complete Cases Sample

eTable 19. Estimated Weekly Mean Daily Step Count Intercepts and Slopes by Age (Within Provinces), Complete Cases Sample

eTable 20. Estimated Weekly Mean Daily Step Count Intercepts and Slopes by Age (Between Provinces), Complete Cases Sample

eTable 21. Estimated Weekly Mean Daily Step Count Intercepts and Slopes by Gender (Within Provinces), Complete Cases Sample

eTable 22. Estimated Weekly Mean Daily Step Count Intercepts and Slopes by Gender (Between Provinces), Complete Cases Sample

eAppendix 4. Code for Primary Data Analysis

This supplemental material has been provided by the authors to give readers additional information about their work.

© 2023 Spilsbury S et al. *JAMA Network Open.*

eTable 1. Behaviour Change Techniques (BCTs)

**Grouping**	**BCTs**
Goals and planning	1.1 Goal setting (behaviour)<br>1.2 Problem solving<br>1.4 Action planning<br>1.8 Behavioural contract<br>1.9 Commitment
Feedback and monitoring	2.1 Monitoring of behaviour by others without feedback<br>2.2 Feedback on behaviour<br>2.3 Self-monitoring of behaviour<br>2.6 Biofeedback
Social support	3.1 Social support (unspecified)
Shaping knowledge	4.2 Information about antecedents
Natural consequences	5.1 Information about health consequences
Associations	7.1 Prompts/cues<br>7.2 Cue signalling reward
Repetition and substitution	8.3 Habit formation<br>8.7 Graded tasks
Reward and threat	10.1 Material incentive (behaviour)
Scheduled consequences	14.9 Reduce reward frequency

© 2023 Spilsbury S et al. *JAMA Network Open.*

eTable 2. Financial Incentive Design Features and Attributes

**Features**	**Attribute**
Form	Consumer loyalty points
Magnitude	\$0.04 per day (35% achievement rate) + \$0.40 per week (2.5% achievement rate)
Target	Personalized and adaptive daily step count (i.e., every two weeks new personalized daily step goal calculated using the median daily step count from the previous 30 days plus 10%)
Timing of assessment	Set intervals (i.e., daily and weekly)
Type of assessment	Objective (i.e., smartphone accelerometer)
Reward immediacy	Instantaneous
Certainty	Certain
Schedule	Indexed
Dispensing type	Resetting
Participant investment	Opportunity cost only (time)
Information disclosure	Factual
Duration	More than 12 months
Source	Government
Recipient	Individual and group

© 2023 Spilsbury S et al. *JAMA Network Open.*

eAppendix 1. Carrot Rewards mHealth App “Steps” Feature Overview

For a personalized “Steps” goal to be generated (ie, steps per day), Carrot Rewards users must have accumulated at least 5 valid days during an initial 7-day baseline or “run-in” period. A valid day was defined as any day with step counts from 1000 to 40,000, as these numbers were considered reasonable, not outliers. Days with step counts <1000 were considered days smartphones were not worn, and days with step counts above 40,000 were deemed suspiciously high (eg, technology bug) and were excluded. For users with at least 5 valid days, a daily step count average was calculated for the baseline period, with 10% added to set the first daily step goal (rounded to the nearest 100 steps). If users did not have enough valid days (ie, ≤4 days) during the baseline period, a generic 5000 daily step goal was provided until the first and then subsequent bi-monthly adaptive goals were set (ie, using the median daily step count from the previous 30 days plus 10%). The approximate the number of steps taken daily by the average Canadian adult is 5000, as measured by a popular smartphone-based activity tracking app. After the 7-day baseline period, users could begin to earn incentives for reaching or exceeding their individualized daily step goals; a progress wheel illustrated progress for the day. Incentives for daily achievements were worth $0.04 CAD in loyalty points which could be redeemed for consumer goods such as movies or groceries. After 2 weeks of earning daily rewards in the form of points, users could then begin to earn team-based rewards worth $0.40 CAD in points per week for reaching their daily goal ≥10 nonconsecutive times in 7 days with a partner. A bar graph to illustrate team progress was also made available in the app.

© 2023 Spilsbury S et al. *JAMA Network Open.*

eTable 3. Behavioral Economics Principles (ie, Decision Biases) Used in Financial Incentive Interventions

**Leveraged by Carrot Rewards.*

**"Decision Bias"**	**Description**	**Examples**
Present Bias*	Preference for a payoff close to the present time rather than in the future	Incentives (*or information about incentives*) given immediately upon meeting goal
Loss Aversion	Preference to avoid losing something than acquire an equal gain	Information given about incentive lost by failing to meet goal i.e., regret
Over-Optimism	Over-estimate the probability of positive events; individuals think they have a better chance of winning a lottery than they actually do	1-in-5 chance of earning $5 for goal achievement may have a larger effect than a guaranteed $1 reward
Salience*	Information that stands out, is novel, or seems relevant is more likely to affect our actions	Text messages provide timely feedback on incentives earned; variable (mystery) incentives
Herd Behavior*	People do what others are doing instead of making independent decisions	Incentives given to team members only if *all* members meet goal
Commitment	Preference for a consistent self-image, best achieved by making a commitment or pre-commitment	Deposit contract in which own money is lost if fail to meet goal
Fresh Start*	Aspirational behavior around temporal landmarks (e.g., January 1, Mondays)	Incentive for reaching daily step goals five times in a week is reset every Monday
Numerosity*	Tendency for people to equate larger numbers with greater value when comparing currencies of different denominations, even when the actual size is constant	Use of incentives in the form of loyalty points (redeemable for consumer goods) of unclear monetary value

Note: For the purpose of this study, overlapping concepts were grouped together. Present bias included delay discounting and hyperbolic discounting. Loss aversion included endowment effect, regret/anticipated aversion, the IKEA effect and framing. Over-optimism included overweighing of small probabilities. Salience encompassed aspects of mental accounting, and herd behavior aspects of social norms.

© 2023 Spilsbury S et al. *JAMA Network Open.*

eTable 4. Covariate Level Categories and Criteria

Covariate categories	Criteria
Intervention Physical Activity Level[a]	
Physically Inactive	< 5000 steps per day
Moderately Active	5000 – 7499 steps per day
Physically Active	≥ 7500 steps per day
Intervention App Engagement[b]	
Low	0 ≤ 4 weeks
Medium	5 ≤ 8 weeks
High	9 ≤ 12 weeks
Total App Experience[c]	
Low	< 6 months
Medium	6 ≤ 12 months
High	> 12 months
Gender[d]	
Female	Socialized gender identification
Male	Socialized gender identification
Other	Socialized gender identification
Age[d]	
13 – 17	Years
18 – 25	Years
26 – 35	Years
36 – 45	Years
46 – 55	Years
56 – 65	Years
66 – 75	Years
76 – 85	Years
> 85	Years

Note. [a] = average of weekly mean daily step count during the intervention period; [b] = number of weeks with at least one application opening during the intervention period; [c] = number of months prior to week 12 that the “Steps” program was enabled; [d] = self-reported

eAppendix 2. Multiple Linear Regression Model, Primary Analysis

$$\gamma_t = \beta_0 + \beta_1 T + \beta_2 X_2 + \ldots + \beta_7 X_7 + \beta_8 TX_2 + \ldots + \beta_{13} TX_7 + \beta_{14} Gender_i + \ldots + \beta_{17} Age + \beta_{18} Household\ Income_i + \ldots + \beta_{28} Loyalty\ Rewards\ Program_i + \ldots + \beta_{34} Baseline\ Step\ Count + \beta_{35} Intervention\ App\ Experience + \beta_{36} Intervention\ App\ Engagement + \beta_{37} Intervention\ Level\ of\ Physical\ Activity$$

Where T represented the number of weeks since the start of the study and γ_t was weekly mean daily step count at time t. X_i was a seven-level categorical variable indicating the post-intervention period (i.e., X_1 = intervention; $X_2 – X_6$ = Winter Holiday Period weeks 13, 14, 15, 16, and 17; X_7 = post-intervention). The regression coefficients β_0, β_1, $\beta_1 + \beta_{8-12}$, and $\beta_1 + \beta_{13}$ represented weekly mean daily step count at $T = 0$ and slope during the intervention, Winter Holiday, and post-intervention periods, respectively. The additive effects of all covariates are indicated by $\beta_{14–37}$, where categorical covariates were represented by i and the number of levels equaled the sum of regression coefficients assigned to each variable (i.e., $\beta_{14}Gender_1$ = female, $\beta_{15}Gender_2$ = male, $\beta_{16}Gender_3$ = other). Intervention app experience, app engagement and level of PA along with age were included as continuous covariates.

© 2023 Spilsbury S et al. *JAMA Network Open.*

eTable 5. Intervention Period Behaviours, Complete Cases (CC) Sample

Variable	Ontario (n = 278,146)	British Columbia (n = 47,410)	Newfoundland and Labrador (n = 12,469)
App Engagement[a] *(weeks; mean ± SD)*[b]	$9.37 \pm 3.74_a$	10.11 ± 3.17*	$9.38 \pm 3.71_a$
App Experience[c] *(months; mean ± SD)*[b]	12.75 ± 5.57*	$17.54 \pm 8.45_b$	$17.93 \pm 8.93_b$
Physical Activity (weekly mean daily step count; mean ± SD)[b]	6431 ± 3058*	6712 ± 3181*	5863 ± 3124*

Note. *SD* = standard deviation. Means sharing a common subscript were not significantly different at $p < .05$ according to the Independent-Samples Kruskal-Wallis test.

[a] = weeks the app was opened at least once during the intervention period (Weeks 1 – 12). [b] = Independent-Samples Kruskal-Wallis Test; [c] = months since "Steps" feature enabled prior to Week 12.

*Between province difference, $p < .05$.

© 2023 Spilsbury S et al. *JAMA Network Open.*

eTable 6. Baseline Characteristics, Multiple Imputation Sample

Variable	Ontario (n = 438,731)	British Columbia (n = 124,101)	Newfoundland and Labrador (n = 21,928)
Age (mean ± SD)[a]	30.89 ± 15.51[*]	32.78 ± 15.80$_a$	32.78 ± 15.16$_a$
Gender[b]			
Female	276,240 (63.0%)[*]	79,611 (64.1%)[*]	15,579 (71.0%)[*]
Male	156,233 (35.6%)[*]	42,185 (34.0%)[*]	6110 (27.9%)[*]
Other	6258 (1.4%)[*]	2305 (1.9%)[*]	239 (1.1%)[*]
Household Income (CAD/year)[b]			
< 20,000	33,773 (7.7%)[*]	4831 (3.9%)[*]	1467 (6.7%)
20,000 > 40,000	45,997 (10.5%)[*]	7449 (6.0%)[*]	1984 (9.0%)
40,000 > 60,000	54,928 (12.5%)[*]	9643 (7.8%)[*]	2272 (10.4%)[*]
60,000 > 80,000	46,020 (10.5%)[*]	7759 (6.3%)[*]	1935 (8.8%)[*]
80,000 > 100,000	37,526 (8.6%)[*]	6091 (4.9%)[*]	1773 (8.1%)
100,000 > 150,000	40,476 (9.2%)[*]	6864 (5.5%)[*]	2188 (10.0%)[*]
≥ 150,000	28,637 (6.5%)[*]	3859 (3.1%)[*]	1403 (6.4%)[*]
Didn't Complete Survey	4023 (0.9%)[*]	416 (0.3%)[*]	154 (0.7%)
Don't Know	14,107 (3.2%)[*]	1823 (1.5%)[*]	493 (2.2%)[*]
Rather Not Say	52,209 (11.9%)[*]	9207 (7.4%)[*]	2560 (11.7%)[*]
NA	81,035 (18.5%)[*]	66,159 (53.3%)[*]	5699 (26.0%)
Loyalty Rewards Program[b]			
Aeroplan® Miles	63,553 (14.5%)[*]	20,053 (16.2%)[*]	5891 (26.9%)[*]
Drop Points	19,512 (4.4%)[*]	3799 (3.1%)[*]	979 (4.5%)
More Rewards®	726 (0.2%)[*]	16,857 (13.6%)[*]	57 (0.2%)[*]
Petro-Points™	50,187 (11.4%)[*]	7913 (6.4%)[*]	150 (0.7%)[*]
RBC Rewards®	9152 (2.1%)[*]	3199 (2.5%)[*]	341 (1.6%)[*]
SCENE Points®	295,601 (67.4%)[*]	72,280 (58.2%)[*]	14,510 (66.1%)
Baseline step count[c] *(steps/day; mean ± SD)*[a]	5780 ± 3818[*]	5922 ± 3636[*]	5283 ± 3435[*]

Note. *SD* = standard deviation. *CAD* = Canadian dollars. Means sharing a common subscript were not significantly different at $p < .05$ according to the Independent-Samples Kruskal-Wallis test. [a] = Independent-Samples Kruskal-Wallis Test; [b] = Chi-squared test of independence; [c] = mean daily step count 14-days prior to Week 1. [*] = $p < .05$.

© 2023 Spilsbury S et al. *JAMA Network Open.*

eTable 7. Baseline Characteristics of Users Excluded From the Complete Cases Sample

Variable	Ontario (n = 160,585)	British Columbia (n = 76,691)	Newfoundland and Labrador (n = 9459)
Age (years; mean ± SD)[a]	33.70 ± 12.95*†	34.88 ± 12.94$_a$†	34.41 ± 12.43$_a$†
Gender[b]			
Female	96,496 (60.1%)*†	47,927 (62.5%)*†	6619 (70.0%)*†
Male	61,868 (38.5%)*†	27,401 (35.7%)*†	2712 (28.7%)*
Other	2221 (0.5%)*	1363 (1.8%)*	128 (1.4%)†
Household Income (CAD/year)[c]			
< 20,000	7877 (4.9%)*†	963 (1.3%)*†	384 (4.1%)†
20,000 > 40,000	11,422 (7.1%)*	1530 (2.0%)*†	510 (5.4%)†
40,000 > 60,000	12,746 (7.9%)*†	1866 (2.4%)*†	547 (5.8%)†
60,000 > 80,000	10,059 (6.3%)*†	1384 (1.8%)*†	441 (4.7%)†
80,000 > 100,000	8255 (5.1%)*†	1052 (1.4%)*†	399 (4.2%)†
100,000 > 150,000	8897 (5.5%)*†	1123 (1.5%)*†	483 (5.1%)*†
≥ 150,000	5607 (3.5%)*†	538 (0.7%)*†	283 (3.0%)†
Didn't Complete Survey	803 (0.5%)*†	79 (0.1%)*†	33 (0.3%)†
Don't Know	2935 (1.8%)*†	353 (0.5%)*†	127 (1.3%)†
Rather Not Say	10,949 (6.8%)*†	1644 (2.1%)*†	553 (5.8%)†
NA	81,035 (50.5%)*†	66,159 (86.3%)*†	5699 (60.2%)†
Loyalty Rewards Program[d]			
Aeroplan® Miles	22,469 (14.0%)*†	11,458 (14.9%)†	2571 (27.2%)*
Drop Points	6907 (4.3%)*†	2184 (2.8%)*†	381 (4.0%)
More Rewards®	325 (0.2%)*†	12,001 (15.6%)*†	35 (0.4%)*
Petro-Points™	19,943 (12.4%)*†	4924 (6.4%)*	63 (0.7%)*
RBC Rewards®	3428 (2.1%)*	2159 (2.8%)*†	129 (1.4%)*
SCENE Points®	107,513 (67.0%)*†	43,965 (57.3%)*†	6280 (66.4%)*
Baseline step count[e] *(steps/day; mean ± SD)*[a]	5831 ± 3986*	5946 ± 3710*	5252 ± 3367*

Note. *SD* = standard deviation. *CAD* = Canadian dollars. Means sharing a common subscript were not significantly different between provinces at $p < .05$ according to the Independent-Samples Kruskal-Wallis test.

[a] = Independent-Samples Kruskal-Wallis Test; [b] = Chi-squared test of independence;
[c] = mean daily step count over 14-days prior to Study Week 1.

© 2023 Spilsbury S et al. *JAMA Network Open.*

eTable 8. Intervention Period Behaviours of Users Excluded From the Complete Cases Sample

Variable	Ontario (n = 160,585)	British Columbia (n = 76,691)	Newfoundland and Labrador (n = 9459)
App Engagement[a] *(weeks; mean ± SD)*[b]	5.92 ± 4.86[*†]	7.04 ± 4.78[*†]	5.56 ± 4.76[*†]
App Experience[c] *(months; mean ± SD)*[b]	11.41 ± 5.71[*†]	14.68 ± 8.49[*†]	19.72 ± 8.77[*†]
Level of Physical Activity (weekly mean daily step count; mean ± SD)[b]	6310 ± 2982[*†]	6387 ± 2924[*†]	5685 ± 2967[*†]

Note. *SD* = standard deviation. Means sharing a common subscript were not significantly different between provinces at $p < .05$ according to the Independent-Samples Kruskal-Wallis test.
[a] = weeks the app was opened at least once during the pre-intervention period (Study Weeks 1 – 12). [b] = Independent-Samples Kruskal-Wallis Test; [c] = months since “Steps” program enabled prior to Week 12.
[*] = $p < .05$ between provinces; [†] = $p < .05$ between analytic sample and excluded participants.

© 2023 Spilsbury S et al. *JAMA Network Open.*

eTable 9. Estimated Weekly Mean Daily Step Count Slopes (Within Provinces), Complete Cases (CC) Sample

**Parameter**	**Ontario**			**British Columbia**			**Newfoundland and Labrador**		
	$\hat{B}$	***SE***	**95% CI**	$\hat{B}$	***SE***	**95% CI**	$\hat{B}$	***SE***	**95% CI**
Slope									
Intervention[a]	-63	-0.47	[-64, -62]	-42	1.20	[-44, -40]	-62	2.21	[-66, -58]
Post-interv.[b]	8*	0.82	[7, 10]	-4*	-1.93	[-8, -1]	-2	3.52	[-9, 5]

Note. $\hat{B}$ = unstandardized regression coefficient; *SE* = robust standard error; CI = confidence interval.
[a] = Weeks 1 - 12; [b] = Weeks 18 – 25; interv. = intervention.
[*] p < .001

© 2023 Spilsbury S et al. *JAMA Network Open.*

eAppendix 3. Sensitivity Analysis Results Summary

Within province estimates of the intervention and post-intervention intercept for the MI sample using multiply imputed data are presented in Supplement 9. Consistent with the CC analysis, intervention to post-intervention estimated intercepts dropped in ON and BC (-159 steps/d and -89 steps/d, respectively). The PA decline in NL, on the other hand, was not statistically significant (-40 steps/d). Furthermore, the intervention to post-intervention intercept difference was greatest when comparing ON to BC and NL (-70 steps/d and -119 steps/d, respectively). Notably, the difference between BC and NL was not statistically significant (40 steps/d). In terms of weekly mean daily step count slopes, statistically significant post-intervention slopes were observed in ON (6 steps/wk) and BC (-6 steps/wk), but not NL (-3 steps/wk), though the rates of change were modest in terms of steps per day.

© 2023 Spilsbury S et al. *JAMA Network Open.*

eTable 10. Estimated Weekly Mean Daily Step Count Intercepts and Slopes (Within Provinces), Multiple Imputation Sample

**Parameter**	**Ontario**			**British Columbia**			**Newfoundland and Labrador**		
	$\hat{B}$	***SE***	**95% CI**	$\hat{B}$	***SE***	**95% CI**	$\hat{B}$	***SE***	**95% CI**
Intercept									
Intervention[a]	6070**	4.062	[6062, 6078]	6319**	7.496	[6304, 6333]	5555**	18.85	[5518, 5592]
Post-[b]	5911**	3.872	[5904, 5919]	6230**	7.920	[6215, 6246]	5515**	17.95	[5479, 5550]
Difference	159	5.60	[148, 170]	89	10.90	[68, 110]	40	26.00	[-11, 91]
Slope									
Intervention[c]	-48.19**	0.333	[-48.84, -47.54]	-31.79**	0.665	[-33.09, -30.49]	-41.33**	1.598	[-44.46, -38.20]
Post-[d]	5.941**	0.667	[4.63, 7.25]	-5.551**	1.049	[-7.61, -3.50]	-2.810	2.989	[-8.67, 3.05]

Note. $\hat{B}$ = unstandardized regression coefficient; *SE* = robust standard error; CI = confidence interval.
[a] = week 12; [b] = week 21; [c] = weeks 1 - 12; [d] = weeks 18 - 25.
* = $p < .05$; ** = $p < .01$.

© 2023 Spilsbury S et al. *JAMA Network Open.*

eTable 11. Estimated Weekly Mean Daily Step Count Intercepts and Slopes (Between Provinces), Multiple Imputation Sample

**Parameter**	**Ontario and British Columbia**			**Ontario and Newfoundland and Labrador**			**British Columbia and Newfoundland and Labrador**		
	$\hat{B}$	***SE***	**95% CI**	$\hat{B}$	***SE***	**95% CI**	$\hat{B}$	***SE***	**95% CI**
Intercept									
Intervention[a]	-248.7**	8.526	[-265.4, -232.0]	514.7**	19.28	[476.9, 552.4]	763.4**	20.28	[723.6, 803.1]
Post-[b]	-318.9**	8.816	[-336.2, -301.6]	396.8**	18.36	[360.8, 432.8]	715.7**	19.62	[677.2, 754.1]
Slope									
Intervention[c]	-16.40**	0.744	[-17.86, -14.94]	-6.863**	1.632	[-10.06, -3.66]	9.536**	1.731	[6.14, 12.93]
Post-[d]	11.49**	1.243	[9.06, 13.93]	8.751**	3.063	[2.75, 14.75]	-2.741	3.168	[-8.95, 3.47]

Note. $\hat{B}$ = unstandardized regression coefficient; *SE* = robust standard error; CI = confidence interval.
[a] = week 12; [b] = week 21; [c] = weeks 1 - 12; [d] = weeks 18 - 25.
* = $p < .05$; ** = $p < .01$.

© 2023 Spilsbury S et al. *JAMA Network Open.*

eTable 12. Difference of the Intervention to Postintervention Weekly Mean Daily Step Count Change (Between Province), Multiple Imputation Sample

**Parameter**	**Ontario vs. British Columbia**			**Ontario vs. Newfoundland and Labrador**			**British Columbia vs. Newfoundland and Labrador**		
	$\hat{B}$	***SE***	**95% CI**	$\hat{B}$	***SE***	**95% CI**	$\hat{B}$	***SE***	**95% CI**
Difference-in-differences[a]	70*	13.21	[46, 94]	119*	24.80	[67, 171]	49*	28.2	[-6, 104]

Note. $\hat{B}$ = unstandardized regression coefficient; *SE* = unpooled standard error; CI = confidence interval.
[a]Difference in differences = Difference of the within province intervention to post-intervention weekly mean daily step count differences
[*]Confidence interval indicates statistical significance (as it does not include zero).

© 2023 Spilsbury S et al. *JAMA Network Open.*

eTable 13. Estimated Weekly Mean Daily Step Count Intercepts and Slopes by Application Engagement (Within Provinces), Complete Cases Sample

Parameter	Ontario			British Columbia			Newfoundland and Labrador		
	$\widehat{B}$	SE	95% CI	$\widehat{B}$	SE	95% CI	$\widehat{B}$	SE	95% CI
Intercept									
Intervention[a]									
Low[b]	6263**	11.84	[6239, 6286]	6636**	40.47	[6556, 6715]	5622**	52.40	[5519, 5725]
Medium[b]	6255**	10.35	[6235, 6275]	6629**	35.95	[6559, 6700]	5632**	45.83	[5543, 5722]
High[b]	6149**	4.117	[6141, 6157]	6526**	10.35	[6506, 6546]	5552**	18.11	[5517, 5588]
Post-[c]									
Low[b]	6052**	19.13	[6015, 6090]	6704**	52.28	[6601, 6806]	5742**	88.63	[5568, 5916]
Medium[b]	6036**	16.74	[6003, 6069]	6680**	46.48	[6589, 6771]	5719**	77.68	[5566, 5871]
High[b]	5821**	6.547	[5808, 5833]	6400**	13.69	[6373, 6427]	5520**	29.60	[5462, 5578]
Slope									
Intervention[d]									
Low[b]	-46.63**	1.608	[-49.78, -43.48]	-20.81**	5.482	[-31.56, -10.07]	-52.55**	7.971	[-68.17, -36.92]
Medium[b]	-60.44**	1.436	[-63.25, -57.62]	-39.25**	3.987	[-47.06, -31.43]	-46.17**	6.458	[-58.83, -33.52]
High[b]	-64.55**	0.516	[-65.56, -63.54]	-43.35**	1.279	[-45.86, -40.85]	-64.82**	2.419	[-69.56, -60.08]

(continued)

© 2023 Spilsbury S et al. *JAMA Network Open.*

Parameter	Ontario			British Columbia			Newfoundland and Labrador		
	$\hat{B}$	SE	95% CI	$\hat{B}$	SE	95% CI	$\hat{B}$	SE	95% CI
Slope									
Post-[e]									
Low[b]	24.23**	4.417	[15.58, 32.89]	6.018	11.44	[-16.41, 28.45]	8.503	18.16	[-27.09, 44.10]
Medium[b]	10.99**	3.471	[4.19, 17.80]	4.828	7.669	[-10.20, 19.86]	1.691	14.57	[-26.87, 30.25]
High[b]	7.538**	0.860	[5.85, 9.22]	-5.512*	2.018	[-9.47, -1.56]	-3.004	3.688	[-10.23, 4.23]

Note. $\hat{B}$ = unstandardized regression coefficient; SE = robust standard error; CI = confidence interval.

[a] = study week 12; [b] = 0 ≤ 4, 5 ≤ 8, and 9 ≤ 12 weeks the application was opened at least once during intervention period for low, medium, and high engagement, respectively; [c] = study week 21; [d] = study weeks 1 - 12; [e] = study weeks 18 - 25.

* = p < .05; ** = p <.01.

© 2023 Spilsbury S et al. *JAMA Network Open.*

eTable 14. Estimated Weekly Mean Daily Step Count Intercepts and Slopes by Application Engagement (Between Provinces), Complete Cases Sample

Parameter	Ontario and British Columbia			Ontario and Newfoundland and Labrador			British Columbia and Newfoundland and Labrador		
	$\hat{B}$	SE	95% CI	$\hat{B}$	SE	95% CI	$\hat{B}$	SE	95% CI
Intercept									
Intervention[a]									
Low[b]	-373.1**	42.16	[-455.8, -290.5]	640.4**	53.72	[535.1, 745.7]	1014**	66.20	[883.8, 1143]
Medium[b]	-374.5**	37.41	[-447.9, -301.2]	622.5**	46.98	[530.4, 714.6]	997.1**	58.25	[882.9, 1111]
High[b]	-377.3**	11.14	[-399.1, -355.5]	596.3**	18.57	[559.9, 632.7]	973.6**	20.86	[932.7, 1014]
Post-[c]									
Low[b]	-651.7**	55.67	[-760.8, -542.6]	310.5**	90.67	[132.7, 488.2]	962.2**	102.9	[760.5, 1164]
Medium[b]	-643.5**	49.40	[-740.3, -546.7]	317.7**	79.46	[161.9, 473.4]	961.2**	90.52	[783.8, 1139]
High[b]	-579.8**	15.18	[609.5, -550.0]	300.7**	30.32	[241.2, 360.1]	880.4**	32.61	[816.5, 944.3]
Slope									
Intervention[d]									
Low[b]	-25.82**	5.713	[-37.02, -14.62]	5.912	8.132	[-10.03, 21.85]	31.73**	9.674	[12.77, 50.69]
Medium[b]	-21.19**	4.238	[-29.50, -12.88]	-14.26*	6.616	[-27.23, -1.30]	6.926	7.590	[-7.95, 21.80]
High[b]	-21.20**	1.379	[-23.90, -18.49]	0.272	2.473	[-4.58, 5.12]	21.47**	2.736	[16.11, 26.83]

© 2023 Spilsbury S et al. *JAMA Network Open.*

(continued).

Parameter	Ontario and British Columbia			Ontario and Newfoundland and Labrador			British Columbia and Newfoundland and Labrador		
	$\hat{B}$	*SE*	95% CI	$\hat{B}$	*SE*	95% CI	$\hat{B}$	*SE*	95% CI
Slope									
Post-[e]									
Low[b]	18.21	12.27	[-5.83, 42.26]	15.73	18.69	[-20.90, 52.36]	-2.485	21.47	[-44.56, 39.59]
Medium[b]	6.167	8.418	[-10.33, 22.67]	9.304	14.98	[-20.06, 38.67]	3.137	16.47	[-29.14, 35.41]
High[b]	13.05**	2.194	[8.75, 17.35]	10.54*	3.787	[3.12, 17.96]	-2.508	4.204	[-10.75, 5.73]

Note. $\hat{B}$ = unstandardized regression coefficient; *SE* = robust standard error; CI = confidence interval.
[a] = weeks 1 – 12; [b] = 0 ≤ 4, 5 ≤ 8, and 9 ≤ 12 weeks the application was opened at least once pre-intervention for low, medium, and high engagement, respectively; [c] = weeks 18 - 25; [d] = week 12; [e] = week 21.
[*] = $p < .05$; [**] = $p < .01$.

© 2023 Spilsbury S et al. *JAMA Network Open.*

eTable 15. Estimated Weekly Mean Daily Step Count Intercepts and Slopes by Intervention Level of Physical Activity (Within Provinces), Complete Cases Sample

Parameter	Ontario			British Columbia			Newfoundland and Labrador		
	$\hat{B}$	*SE*	95% CI	$\hat{B}$	*SE*	95% CI	$\hat{B}$	*SE*	95% CI
Intercept									
Intervention[a]									
Sedentary[b]	3827**	5.658	[3816, 3838]	4025**	13.68	[3998, 4052]	3508**	21.23	[3466, 3549]
Low Active[b]	4504**	4.437	[4496, 4513]	4694**	10.66	[4673, 4715]	4131**	17.81	[4096, 4166]
Physically Active[b]	5469**	4.774	[5460, 5478]	5852**	11.59	[5829, 5874]	4939**	20.41	[4899, 4979]
Post-[c]									
Sedentary[b]	3934**	6.710	[3920, 3947]	4259**	16.30	[4227, 4291]	3695**	25.87	[3644, 3746]
Low Active[b]	4480**	5.183	[4470, 4490]	4818**	12.35	[4794, 4843]	4254**	21.32	[4212, 4295]
Physically Active[b]	5237**	5.700	[5226, 5249]	5805**	13.24	[5779, 5831]	4935**	24.33	[4888, 4983]
Slope									
Intervention[d]									
Sedentary[b]	-38.25**	0.516	[-39.26, -37.24]	-15.89**	1.279	[-18.40, -13.38]	-35.00**	2.096	[-39.11, -30.89]
Low Active[b]	-69.06**	0.777	[-70.58, -67.54]	-43.87**	1.909	[-47.62, -40.13]	-72.91**	4.367	[-58.83, -33.52]
Physically Active[b]	-85.97**	1.189	[-88.31, -83.64]	-65.88**	2.826	[-71.42, -60.34]	-103.3**	6.487	[-116.0, -90.61]

© 2023 Spilsbury S et al. *JAMA Network Open.*

(continued).

Parameter	Ontario			British Columbia			Newfoundland and Labrador		
	$\hat{B}$	*SE*	95% CI	$\hat{B}$	*SE*	95% CI	$\hat{B}$	*SE*	95% CI
Slope									
Post-[e]									
Sedentary[b]	24.75**	1.076	[22.64, 26.86]	13.77**	2.669	[8.54, 19.00]	11.23	3.932	[3.53, 18.94]
Low Active[b]	7.934**	1.309	[5.37, 20.50]	-1.65	2.993	[-7.52, 4.22]	-7.398	6.657	[-20.45, 5.65]
Physically Active[b]	-10.98**	1.957	[-14.82, -7.15]	-24.57*	4.226	[-32.85, -16.29]	-18.20	10.09	[-37.97, 1.56]

Note. $\hat{B}$ = unstandardized regression coefficient; *SE* = robust standard error; CI = confidence interval.
[a] = study week 12; [b] = pre-intervention average of weekly mean daily step count of < 5000, 5000 – 7499, and ≥ 7500 for sedentary, low active, and physically active users, respectively; [c] = study week 21; [d] = study weeks 1 – 12; [e] = study weeks 18 - 25.
* = p < .05; ** = p <.01.

© 2023 Spilsbury S et al. *JAMA Network Open.*

eTable 16. Estimated Weekly Mean Daily Step Count Intercepts and Slopes by Preintervention Level of Physical Activity (Between Provinces), Complete Cases Sample

Parameter	Ontario and British Columbia			Ontario and Newfoundland and Newfoundland and Labrador			British Columbia and Newfoundland and Labrador		
	$\hat{B}$	*SE*	95% CI	$\hat{B}$	*SE*	95% CI	$\hat{B}$	*SE*	95% CI
Intercept									
Intervention[a]									
Sedentary[b]	-197.6**	14.80	[-226.7, -168.6]	319.6**	21.98	[276.6, 362.7]	517.3**	25.26	[467.8, 566.8]
Low Active[b]	-189.7**	11.54	[-212.4, -167.1]	373.7**	18.35	[337.7, 409.7]	563.4**	20.75	[522.8, 604.1]
Physically Active[b]	-382.6**	12.54	[-407.2, -358.1]	530.5**	20.96	[489.4, 571.6]	913.1**	23.48	[867.1, 959.1]
Post-[c]									
Sedentary[b]	-325.4**	17.62	[-360.0, -290.9]	238.7**	26.73	[186.3, 291.1]	564.1**	30.57	[504.2, 624.0]
Low Active[b]	-338.1**	13.39	[-364.3, -311.8]	226.6**	13.39	[200.4, 252.9]	564.7**	24.64	[516.4, 613.0]
Physically Active[b]	-568.1**	14.42	[-596.3, -539.8]	302.0**	24.99	[253.0, 350.9]	870.0**	27.70	[815.8, 924.3]
Slope									
Intervention[d]									
Sedentary[b]	-22.36**	1.379	[-25.06, -19.66]	-3.246	2.159	[-7.48, -0.99]	19.11**	2.455	[14.30, 23.93]
Low Active[b]	-25.19**	2.061	[-29.23, -21.15]	3.848	4.436	[-4.85, 12.54]	29.04**	4.766	[19.69, 38.38]
Physically Active[b]	-20.10**	3.066	[-26.11, -14.09]	17.35	6.595	[4.42, 30.27]	37.44**	7.076	[23.57, 51.31]

© 2023 Spilsbury S et al. *JAMA Network Open.*

(continued).

Parameter	Ontario and British Columbia			Ontario and Newfoundland and Labrador			British Columbia and Newfoundland and Labrador		
	**$\hat{B}$**	***SE***	**95% CI**	**$\hat{B}$**	***SE***	**95% CI**	**$\hat{B}$**	***SE***	**95% CI**
Slope									
Post-[e]									
Sedentary[b]	10.98**	2.878	[5.34, 16.62]	13.52**	4.077	[5.53, 21.51]	2.536	4.752	[-6.78, 11.85]
Low Active[b]	9.584**	3.267	[3.18, 15.99]	15.33*	6.784	[2.04, 28.63]	5.748	7.299	[-8.56, 20.05]
Physically Active[b]	13.59**	4.657	[4.46, 22.71]	7.219	10.27	[-12.92, 27.35]	-6.368	10.94	[-27.80, 15.06]

Note. $\hat{B}$ = unstandardized regression coefficient; *SE* = robust standard error; CI = confidence interval.
[a] = study week 12; [b] = pre-intervention average of weekly mean daily step count of < 5000, 5000 – 7499, and ≥ 7500 for sedentary, low active, and physically active users, respectively; [c] = study week 21; [d] = study weeks 1 – 12; [e] = study weeks 18 - 25.
* = p < .05; ** = p <.01.

© 2023 Spilsbury S et al. *JAMA Network Open.*

eTable 17. Estimated Weekly Mean Daily Step Count Intercepts and Slopes by Application Experience (Within Provinces), Complete Cases Sample

Parameter	Ontario			British Columbia			Newfoundland and Labrador		
	$\hat{B}$	*SE*	95% CI	$\hat{B}$	*SE*	95% CI	$\hat{B}$	*SE*	95% CI
Intercept									
Intervention[a]									
Low[b]	6064**	7.557	[6050, 6079]	6457**	23.68	[6411, 6504]	5425**	43.80	[5340, 5511]
Medium[b]	6093**	5.466	[6082, 6104]	6473**	18.56	[6436, 6509]	5455**	34.06	[5388, 5521]
High[b]	6125**	4.198	[6117, 6133]	6520**	9.764	[6501, 6539]	5545**	18.66	[5508, 5581]
Post-[c]									
Low[b]	5679**	11.59	[5657, 5702]	6310**	31.44	[6248, 6372]	5285**	64.77	[5158, 5412]
Medium[b]	5712**	8.265	[5696, 5728]	6324**	24.61	[6276, 6372]	5332**	50.14	[5234, 5431]
High[b]	5754**	6.250	[5742, 5767]	6352**	12.87	[6327, 6378]	5432**	26.58	[5380, 5484]
Slope									
Intervention[d]									
Low[b]	-77.75**	1.258	[-80.21, -75.28]	-56.84**	4.139	[-64.96, -48.73]	-90.36**	7.592	[-105.2, -75.48]
Medium[b]	-63.39**	0.816	[-64.99, -61.79]	-46.29**	2.404	[-51.01, -41.58]	-66.38**	4.597	[-75.39, -57.37]
High[b]	-57.87**	0.642	[-59.12, -56.61]	-38.05**	1.458	[-40.91, -35.19]	-56.36**	2.648	[-61.55, -51.17]

© 2023 Spilsbury S et al. *JAMA Network Open.*

(continued).

Parameter	Ontario			British Columbia			Newfoundland and Labrador		
	$\hat{B}$	*SE*	95% CI	$\hat{B}$	*SE*	95% CI	$\hat{B}$	*SE*	95% CI
Slope									
Post-[e]									
Low[b]	2.894	2.238	[-1.49, 7.28]	-11.05	6.158	[-23.12, 1.02]	13.51	11.94	[-9.90, 36.91]
Medium[b]	6.416**	1.445	[3.59, 9.25]	-7.260	3.956	[-15.01, 0.49]	-6.314	7.333	[-20.69, 8.06]
High[b]	10.85**	1.119	[8.65, 13.04]	-2.225	2.361	[2.40, -0.94]	-2.847	4.255	[-11.19, 5.49]

Note. $\hat{B}$ = unstandardized regression coefficient; *SE* = robust standard error; CI = confidence interval.
[a] = study week 12; [b] = < 6, 6 ≤ 12, and > 12 months between the date that the "Steps" program was enabled and week 12 for low, medium, and high experience, respectively; [c] = study week 21; [d] = study weeks 1 - 12; [e] = study weeks 18 - 25.
* = $p < .05$; ** = $p < .01$.

© 2023 Spilsbury S et al. *JAMA Network Open.*

eTable 18. Estimated Weekly Mean Daily Step Count Intercepts and Slopes by Application Experience (Between Provinces), Complete Cases Sample

Parameter	Ontario and British Columbia			Ontario and Newfoundland and Labrador			British Columbia and Newfoundland and Labrador		
	$\widehat{B}$	*SE*	95% CI	$\widehat{B}$	*SE*	95% CI	$\widehat{B}$	*SE*	95% CI
Intercept									
Intervention[a]									
Low[b]	-393.0**	24.86	[-441.7, -344.3]	639.0**	44.45	[551.9, 726.1]	1032**	49.80	[934.4, 1130]
Medium[b]	-379.9**	19.35	[-417.8, -342.0]	638.1**	34.50	[570.5, 705.8]	1018**	38.79	[942.0, 1094]
High[b]	-395.1**	10.63	[-416.0, -374.3]	580.4**	19.12	[542.9, 617.8]	975.5**	21.06	[934.2, 1017]
Post-[c]									
Low[b]	-630.7**	33.51	[-696.4, -565.0]	394.3**	65.80	[265.4, 523.3]	1025**	72.00	[883.9, 1166]
Medium[b]	-612.1**	25.96	[-663.0, -561.2]	379.4**	50.81	[279.8, 479.0]	991.5**	55.85	[882.0, 1101]
High[b]	-598.1**	14.30	[-626.2, -570.1]	322.0**	27.30	[268.5, 375.5]	920.1**	29.53	[862.3, 978.0]
Slope									
Intervention[d]									
Low[b]	-20.90**	4.326	[-29.38, -12.42]	12.62	7.696	[-2.47, 27.70]	33.52**	8.647	[16.57, 50.47]
Medium[b]	-17.10**	2.539	[-22.07, -12.12]	2.991	4.669	[-6.16, -12.14]	20.09**	5.188	[9.92, 30.26]
High[b]	-19.82**	1.593	[-22.94, -16.70]	-1.505	2.725	[-6.85, 3.84]	18.31**	3.023	[12.39, 24.24]

© 2023 Spilsbury S et al. *JAMA Network Open.*

(continued).

Parameter	Ontario and British Columbia			Ontario and Newfoundland and Labrador			British Columbia and Newfoundland and Labrador		
	$\hat{B}$	*SE*	95% CI	$\hat{B}$	*SE*	95% CI	$\hat{B}$	*SE*	95% CI
Slope									
Post-[e]									
Low[b]	13.95*	6.552	[1.11, 26.79]	-10.61	12.15	[-34.42, 13.20]	-24.56	13.44	[-50.89, 1.78]
Medium[b]	13.68**	4.212	[5.42, 21.93]	12.73	7.474	[-1.92, 27.38]	-0.946	8.332	[-17.28, 15.38]
High[b]	13.07**	2.613	[7.95, 18.19]	13.69**	4.400	[5.07, 22.32]	0.622	4.866	[-8.92, 10.16]

Note. $\hat{B}$ = unstandardized regression coefficient; *SE* = robust standard error; CI = confidence interval.
[a] = study week 12; [b] = < 6, 6 ≤ 12, and > 12 months between the date that the "Steps" program was enabled and week 12 for low, medium, and high experience, respectively; [c] = study week 21; [d] = study weeks 1 - 12; [e] = study weeks 18 - 25.
* = $p < .05$; ** = $p < .01$.

© 2023 Spilsbury S et al. *JAMA Network Open.*

eTable 19. Estimated Weekly Mean Daily Step Count Intercepts and Slopes by Age (Within Provinces), Complete Cases Sample

**Parameter**	**Ontario**			**British Columbia**			**Newfoundland and Labrador**		
	$\hat{B}$	***SE***	**95% CI**	$\hat{B}$	***SE***	**95% CI**	$\hat{B}$	***SE***	**95% CI**
Intercept									
Intervent.[a]									
13 - 17	6105**	11.11	[6084, 6127]	6415**	35.12	[6346, 6483]	5520**	49.84	[5422, 5618]
18 - 25	6112**	8.233	[6096, 6128]	6431**	28.04	[6376, 6486]	5536**	39.88	[5458, 5614]
26 - 35	6118**	7.895	[6103, 6134]	6453**	24.31	[6405, 6500]	5533**	36.29	[5462, 5604]
36 – 45	6120**	9.164	[6102, 6138]	6447**	28.22	[6391, 6502]	5541**	39.16	[5464, 5617]
46 – 55	6113**	9.828	[6094, 6132]	6430**	30.42	[6371, 6490]	5525**	42.72	[5441, 5609]
56 – 65	6110**	10.48	[6089, 6130]	6431**	32.13	[6368, 6494]	5516**	46.74	[5425, 5608]
66 – 75	6106**	10.97	[6085, 6128]	6416**	34.36	[6348, 6483]	5523**	49.22	[5427, 5620]
76 – 85	6105**	11.10	[6084, 6127]	6415**	35.05	[6346, 6484]	5520**	49.81	[5423, 5618]
> 85	6105**	11.10	[6084, 2127]	6415**	35.09	[6346, 6484]	5520**	49.80	[5422, 5618]
Post-[b]									
13 - 17	5617**	16.48	[5585, 5650]	6367**	57.13	[6255, 6479]	5510**	80.68	[5351, 5668]
18 - 25	5677**	12.15	[5653, 5700]	6370**	45.41	[6281, 6459]	5502**	63.73	[5377, 5627]
26 - 35	5671**	11.67	[5648, 5694]	6362**	39.30	[6285, 6439]	5507**	58.43	[5392, 5621]
36 – 45	5652**	13.60	[5625, 5679]	6373**	45.84	[6283, 6463]	5502**	63.05	[5378, 5625]
46 – 55	5633**	14.59	[5604, 5661]	6374**	49.44	[6277, 6470]	5498**	68.94	[5363, 5634]
56 – 65	5622**	15.56	[5591, 5652]	6358**	52.25	[6256, 6460]	5505**	75.63	[5357, 5654]
66 – 75	5619**	16.28	[5587, 5651]	6364**	55.89	[6254, 6473]	5515**	79.68	[5359, 5671]
76 - 85	5618**	16.47	[5585, 5650]	6366**	57.02	[6255, 6478]	5510**	80.64	[5352, 5668]
> 85	5618**	16.48	[5585, 5650]	6366**	57.09	[6254, 6478]	5510**	80.63	[5352, 5668]

© 2023 Spilsbury S et al. *JAMA Network Open.*

(continued).

Parameter	Ontario			British Columbia			Newfoundland and Labrador		
	$\hat{B}$	*SE*	95% CI	$\hat{B}$	*SE*	95% CI	$\hat{B}$	*SE*	95% CI
Slope									
Intervent.[c]									
13 - 17	-66.21**	1.902	[-69.94, -62.48]	-54.00**	6.050	[-65.86, -42.15]	-62.44**	8.239	[-78.59, -46.29]
18 - 25	-65.07**	0.850	[-66.74, -63.41]	-46.83**	2.464	[-51.66, -42.00]	-53.60**	4.342	[-62.11, -45.09]
26 - 35	-62.23**	0.876	[-64.95, -61.52]	-40.68**	2.158	[-44.91, -36.45]	-62.11**	3.998	[-69.95, -54.28]
36 – 45	-58.85**	1.068	[-60.94, -56.75]	-36.60**	2.491	[-41.49, -31.72]	-60.49**	4.536	[-69.38, -51.60]
46 – 55	-62.30**	1.390	[-65.02, -59.57]	-45.57**	3.402	[-52.24, -38.90]	-69.88**	6.301	[-82.23, -57.53]
56 – 65	-62.78**	2.146	[-66.98, -58.57]	-35.65**	4.600	[-44.67, -26.64]	-83.76**	9.920	[-103.2, -64.31]
66 – 75	-52.95**	4.411	[-61.60, -44.31]	-55.01**	8.176	[-71.03, -38.99]	-26.12	19.77	[-64.87, 12.64]
76 – 85	-40.04**	12.55	[-64.64, -15.44]	-13.27	32.44	[-76.85, 50.32]	68.36	73.77	[-76.21, 212.9]]
> 85	-34.72	18.11	[-70.21, 0.77]	-15.20	32.96	[-79.79, 49.40]	-85.88	54.61	[-192.9, 21.16]
Post-[d]									
13 - 17	-10.02*	3.747	[-17.37, -2.68]	-5.609	11.15	[-27.45, 16.24]	-37.98*	15.81	[-68.98, -6.98]
18 - 25	-3.025	1.568	[-6.10, 0.05]	-3.981	4.262	[-12.34, 4.37]	-20.24*	7.360	[-34.67, -5.82]
26 - 35	18.75**	1.524	[15.77, 21.74]	-0.906	3.404	[-7.58, 5.77]	11.27	6.665	[-1.80, 24.33]
36 – 45	9.671**	1.850	[6.04, 13.30]	2.708	4.057	[-5.24, 10.66]	-2.049	7.136	[-16.04, 11.94]
46 – 55	9.476**	2.326	[4.92, 14.03]	-10.86	5.235	[-21.12, -0.60]	-7.537	9.037	[-25.25, 10.18]
56 – 65	14.37**	3.532	[7.45, 21.30]	-16.27	7.160	[-30.30, -2.23]	10.03	14.07	[-17.55, 37.62]
66 – 75	11.29	7.322	[-3.064, 25.64]	-26.21	13.03	[-51.74, -0.68]	75.27	42.53	[-8.08, 158.6]
76 – 85	-49.93	26.14	[-101.2, 1.31]	-33.59	42.22	[-116.3, 49.17]	87.77	107.6	[-123.2, 298.7]
> 85	6.821	28.50	[-49.04, 62.68]	23.93	45.29	[-64.83, 112.7]	-52.03	44.97	[-140.2, 36.11]

Note. $\hat{B}$ = unstandardized regression coefficient; *SE* = robust standard error; CI = confidence interval.
[a] = study week 12; [b] = study week 21; [c] = study weeks 1 – 12; [d] = study weeks 18 – 25.
* = $p < .05$; ** = $p < .01$.

© 2023 Spilsbury S et al. *JAMA Network Open.*

eTable 20. Estimated Weekly Mean Daily Step Count Intercepts and Slopes by Age (Between Provinces), Complete Cases Sample

Parameter	Ontario and British Columbia			Ontario and Newfoundland and Labrador			British Columbia and Newfoundland and Labrador		
	$\hat{B}$	*SE*	95% CI	$\hat{B}$	*SE*	95% CI	$\hat{B}$	*SE*	95% CI
Intercept									
Intervent.[a]									
13 - 17	-309.3**	36.83	[-384.4, -237.2]	585.4**	51.06	[485.3, 685.4]	894.6**	60.97	[775.1, 1014]
18 - 25	-318.4**	29.22	[-375.7, -261.2]	575.9**	40.72	[496.1, 655.7]	894.4**	48.74	[798.8, 989.9]
26 - 35	-334.6**	25.26	[-384.7, -284.5]	584.8**	37.14	[512.0, 657.6]	919.4**	43.68	[833.8, 1005]
36 – 45	-326.3**	29.67	[-384.5, -268.2]	579.7**	40.22	[500.8, 658.5]	906.0**	48.27	[811.4, 1001]
46 – 55	-317.5**	31.97	[-380.1, -254.8]	587.9**	43.83	[502.0, 673.8]	905.3**	52.44	[802.6, 1008]
56 – 65	-321.4**	33.80	[-387.6, -255.1]	593.4**	47.90	[499.5, 687.2]	914.7**	56.72	[803.6, 1026]
66 – 75	-309.1**	36.07	[-379.8, -238.4]	583.1**	50.43	[484.2, 681.9]	892.2**	60.03	[774.5, 1010]
76 – 85	-309.7**	36.76	[-381.7, -237.6]	585.0**	51.03	[485.0, 685.0]	894.7**	60.91	[775.3, 1014]
> 85	-309.3**	36.81	[-381.5, -237.2]	585.6**	51.03	[485.6, 685.6]	894.9**	60.93	[775.5, 1014]
Post-[b]									
13 - 17	-749.4**	59.46	[-866.0, -632.9]	107.8	82.35	[-53.63, 269.2]	857.2**	98.86	[663.4, 1051]
18 - 25	-693.1**	47.00	[-785.2, -600.9]	174.8*	64.88	[47.58, 301.9]	867.8**	78.25	[714.4, 1021]
26 - 35	-691.3**	40.99	[-771.6, -610.9]	164.3*	59.59	[47.48, 281.1]	855.6**	70.42	[717.5, 993.6]
36 – 45	-721.0**	47.81	[-814.8, -627.3]	150.3*	64.50	[23.93, 276.8]	871.4**	77.95	[718.6, 1024]
46 – 55	-740.7**	51.55	[-841.7, -639.7]	134.4	70.46	[-3.72, 272.5]	875.1**	84.83	[708.8, 1041]
56 – 65	-736.5**	54.52	[-843.4, -629.7]	116.0	77.21	[-35.30, 267.4]	852.6**	91.92	[672.4, 1033]
66 – 75	-745.0**	58.21	[-859.1, -630.9]	104.1	81.32	[-55.32, 263.5]	849.0**	97.33	[658.3, 1040]
76 - 85	-748.9**	59.35	[-865.2, -632.5]	107.4	82.31	[-53.91, 268.7]	856.3**	98.77	[662.7, 1050]

© 2023 Spilsbury S et al. *JAMA Network Open.*

(continued).

Parameter	Ontario and British Columbia			Ontario and Newfoundland and Labrador			British Columbia and Newfoundland and Labrador		
	$\widehat{B}$	*SE*	95% CI	$\widehat{B}$	*SE*	95% CI	$\widehat{B}$	*SE*	95% CI
Intercept									
Post-[b]									
> 85	-748.8**	59.42	[-865.2, -623.3]	108.1	82.29	[-53.23, 269.3]	856.8**	98.79	[663.2, 1050]
Slope									
Pre-[c]									
13 - 17	12.21	6.342	[-24.64, 0.22]	-3.770	8.456	[-20.34, 12.80]	8.438	10.22	[-11.60, 28.47]
18 - 25	-18.24**	2.606	[-23.35, -13.13]	-11.47*	4.424	[-20.14, -2.80]	6.771	4.992	[-3.01, 16.56]
26 - 35	-22.55**	2.329	[-27.12, -17.99]	-1.120	4.093	[-9.14, 6.90]	21.43**	4.543	[12.53, 30.34]
36 – 45	-22.24**	2.710	[-27.55, -16.93]	1.642	4.660	[-7.49, 10.78]	23.88**	5.175	[13.74, 34.03]
46 – 55	-16.73**	3.675	[-23.93, -9.52]	7.585	6.452	[-5.06, 20.23]	24.31**	7.161	[10.28, 38.35]
56 – 65	-27.12**	5.076	[-37.07, 17.18]	20.98*	10.15	[1.09, 40.87]	48.10**	10.94	[26.67, 69.53]
66 – 75	2.059	9.290	[-16.15, 20.27]	-26.84	20.26	[-66.54, 12.87]	-28.89	21.40	[-70.83, 13.04]
76 – 85	-26.77	34.79	[-94.95, 41.41]	-108.4	74.83	[-255.1, 38.25]	-81.63	80.58	[-239.6, 76.31]
> 85	-19.52	37.60	[-93.23, 54.18]	51.16	57.54	[-61.61, 163.9]	70.68	63.79	[-54.34, 195.7]
Post-[d]									
13 - 17	-4.413	11.76	[-27.46, 18.63]	27.96	16.25	[-3.90, 59.81]	32.37	19.35	[-5.55, 70.29]
18 - 25	0.956	4.541	[-7.95, 9.86]	17.22*	7.525	[2.47, 31.97]	16.26	8.505	[-0.41, 32.93]
26 - 35	19.66**	3.730	[12.35, 26.97]	7.483	6.837	[-5.92, 20.88]	-12.17	7.484	[-26.84, 2.49]
36 – 45	6.963	4.459	[-1.78, 15.70]	11.72	7.372	[-2.73, 26.17]	4.757	8.209	[-11.33, 20.85]
46 – 55	20.33**	5.728	[9.11, 31.56]	17.01	9.332	[-1.28, 35.30]	-3.320	10.44	[-23.79, 17.15]
56 – 65	30.64**	7.984	[14.99, 46.28]	4.341	14.51	[-24.10, 32.78]	-26.30	15.79	[-57.24, 4.65]
66 – 75	37.50*	14.94	[8.21, 66.78]	-63.99	43.15	[-148.6, 20.59]	-101.5*	44.48	[-188.7, -14.31]
76 – 85	-16.34	49.66	[-113.7, 80.98]	-137.7	110.8	[-354.8, 79.37]	-121.4	115.6	[-347.9, 105.2]
> 85	-17.11	53.51	[-122.0, 87.77]	58.85	53.24	[-45.50, 163.2]	75.97	63.83	[-49.13, 201.1]

© 2023 Spilsbury S et al. *JAMA Network Open.*

(continued).

Note. $\hat{B}$ = unstandardized regression coefficient; *SE* = robust standard error; CI = confidence interval.
[a] = study week 12; [b] = study week 21; [c] = study weeks 1 – 12; [d] = study weeks 18 – 25.
[*] = $p < .05$; [**] = $p < .01$.

© 2023 Spilsbury S et al. *JAMA Network Open.*

eTable 21. Estimated Weekly Mean Daily Step Count Intercepts and Slopes by Gender (Within Provinces), Complete Cases Sample

Parameter	Ontario			British Columbia			Newfoundland and Labrador		
	$\hat{B}$	*SE*	95% CI	$\hat{B}$	*SE*	95% CI	$\hat{B}$	*SE*	95% CI
Intercept									
Intervention[a]									
Female	6152**	3.537	[6145, 6159]	6528**	8.574	[6511, 6545]	5583**	15.72	[5552, 5614]
Male	6153**	3.062	[6147. 6159]	6535**	7.331	[6521, 6550]	5574**	14.06	[5546, 5602]
Other[b]	6152**	3.510	[6145, 6159]	6527**	8.472	[6511, 6544]	5583**	15.65	[5552, 5614]
Post-[c]									
Female	5776**	5.281	[5765, 5786]	6368**	11.61	[6346, 6391]	5490**	23.19	[5444, 5535]
Male	5786**	4.377	[5777, 5795]	6395**	9.705	[6346, 6384]	5481**	19.84	[5442, 5520]
Other[b]	5776**	5.230	[5765, 5786]	6369**	11.47	[6347, 6392]	5490**	23.06	[5445, 5535]
Slope									
Intervention[d]									
Female	-58.14**	0.555	[-59.22, -57.05]	-39.65**	1.422	[-42.43, -36.86]	-57.03**	2.483	[-61.90, -52.17]
Male	-71.61**	0.879	[-77.33, -69.88]	-46.33**	2.274	[-50.78, -41.87]	-75.80**	4.700	[-85.01, -66.58]
Other[b]	-63.45**	4.006	[-71.31, -55.60]	-48.05**	8.115	[-63.96, -32.15]	-55.08*	20.42	[-95.11, -15.05]

© 2023 Spilsbury S et al. *JAMA Network Open.*

(continued).

Parameter	Ontario			British Columbia			Newfoundland and Labrador		
	$\hat{B}$	*SE*	95% CI	$\hat{B}$	*SE*	95% CI	$\hat{B}$	*SE*	95% CI
Slope									
Post-[e]									
Female	11.69**	0.988	[9.75, 13.62]	-5.893*	2.293	[-10.39, -1.40]	3.571	3.955	[-4.18, 11.32]
Male	1.507	1.505	[-1.44, 4.46]	-2.037	3.635	[-9.16, 5.09]	-17.20*	7.555	[-32.01, -2.39]
Other[b]	14.78*	6.693	[1.66, 27.90]	10.63	13.74	[-16.31, 37.56]	-11.72	31.04	[-72.55, 49.11]

Note. $\hat{B}$ = unstandardized regression coefficient; *SE* = robust standard error; CI = confidence interval.
[a] = study week 12; [b] = identified gender not female or male; [c] = study week 21; [d] = study weeks 1 - 12; [e] = study weeks 18 - 25.
* = $p < .05$; ** = $p < .01$.

© 2023 Spilsbury S et al. *JAMA Network Open.*

eTable 22. Estimated Weekly Mean Daily Step Count Intercepts and Slopes by Gender (Between Provinces), Complete Cases Sample

Parameter	Ontario and British Columbia			Ontario and Newfoundland and Labrador			British Columbia and Newfoundland and Labrador		
	$\widehat{B}$	*SE*	95% CI	$\widehat{B}$	*SE*	95% CI	$\widehat{B}$	*SE*	95% CI
Intercept									
Intervention[a]									
Female	-373.3**	9.275	[-394.5, -358.1]	568.9**	16.11	[537.3, 600.4]	945.2**	17.91	[910.1, 980.3]
Male	-382.2**	7.945	[-397.8, -366.7]	579.1**	14.39	[550.9, 607.4]	961.4**	15.86	[930.3, 992.5]
Other[b]	-375.6**	9.170	[-393.6, -357.6]	568.8**	16.04	[537.3, 600.2]	944.4**	17.80	[909.5, 979.3]
Post-interv.[c]									
Female	-592.8**	12.76	[-617.8, -567.8]	285.8**	23.78	[239.2, 332.4]	878.6**	25.93	[827.8, 929.5]
Male	-578.9**	10.65	[-599.7, -558.0]	305.2**	20.32	[265.4, 345.1]	884.1**	22.09	[840.8, 927.4]
Other[b]	-593.7**	12.61	[-618.4, -569.0]	285.8**	23.65	[239.5, 332.2]	879.5**	25.76	[829.0, 930.0]
Slope									
Intervention[d]									
Female	-18.49**	1.526	[-21.48, 15.50]	-1.102	2.544	[-6.09, 3.89]	17.39**	2.861	[11.78, 22.99]
Male	-25.28**	2.438	[-30.06, -20.50]	4.191	4.781	[-5.18, 13.56]	29.47**	5.221	[19.24, 39.70]
Other[b]	-15.40	9.050	[-33.14, 2.34]	-8.375	20.81	[-49.17, 32.42]	7.025	21.98	[-36.05, 50.10]

© 2023 Spilsbury S et al. *JAMA Network Open.*

(continued).

Parameter	Ontario and British Columbia			Ontario and Newfoundland and Labrador			British Columbia and Newfoundland and Labrador		
	$\hat{B}$	SE	95% CI	$\hat{B}$	SE	95% CI	$\hat{B}$	SE	95% CI
Slope									
Post-[e]									
Female	17.58**	2.497	[12.69, 22.47]	8.116*	4.077	[0.13, 16.11]	-9.464*	4.572	[-18.42, -0.50]
Male	3.544	3.934	[-4.17, 11.26]	18.71*	7.703	[3.61, 33.81]	15.16	8.384	[-1.27, 31.60]
Other[b]	4.155	15.29	[-25.80, 34.11]	26.50	31.75	[-35.73, 88.73]	22.35	33.94	[-44.18, 88.87]

Note. $\hat{B}$ = unstandardized regression coefficient; SE = robust standard error; CI = confidence interval.
[a] = study week 12; [b] = identified gender not female or male; [c] = study week 21; [d] = study weeks 1 - 12; [e] = study weeks 18 - 25.
* = p < .05; ** = p < .01.

© 2023 Spilsbury S et al. *JAMA Network Open.*

eAppendix 4. Code for Primary Data Analysis

```
# Figure 1. Replace X3 with CompleteCases.csv

# Create the Intervention categorical variable with four levels based on the previous exploratory
plot:  weeks 1 - 12, 13, 14, 15, 16, 17 and 18 - 25
X3$Intervention <- "pre"
X3$Intervention[X3$Week == 13] <- "wk13"
X3$Intervention[X3$Week == 14] <- "wk14"
X3$Intervention[X3$Week == 15] <- "wk15"
X3$Intervention[X3$Week == 16] <- "wk16"
X3$Intervention[X3$Week == 17] <- "wk17"
X3$Intervention[X3$Week >= 18] <- "post"
X3$Intervention <- factor(X3$Intervention, levels = levels(factor(X3$Intervention))[c(2, 3, 4, 5,
6, 7, 1)])

lm2 <- lm(Average.Steps ~ Week * NewProvinceColumn * Intervention + gender + age +
current_provider + income_level + first_baseline + Experience_mths + Engagement_pre +
PhysicalActivity_pre, X3)

X3$fit2 <- fitted(lm2)

X3 %>%
  group_by(NewProvinceColumn, Week) %>%
  summarize(Mean = mean(Average.Steps), Fit = mean(fit2)) %>%
  ggplot(aes(x = Week)) +
  geom_line(aes(y = Mean, group = NewProvinceColumn, colour = NewProvinceColumn), size
= 0.2) +
  geom_point(aes(y = Mean, group = NewProvinceColumn, colour = NewProvinceColumn), size
= 2.5) +
  geom_line(aes(y = Fit, group = NewProvinceColumn)) +
  scale_color_discrete(name = "Province",
                       labels = c("British Columbia", "Ontario", "Newfoundland and Labrador")) +
  guides(col = guide_legend(order = 1)) +
  scale_x_continuous(breaks = seq(0, 25, 2)) +
  geom_rect(aes(xmin = 13, xmax = 18, ymin = -Inf, ymax = Inf, fill = "December 2, 2018 -
January 5, 2019"), colour=NA, alpha = .006) +
  scale_fill_manual('Winter Holiday Period (excluded)', values = 'gray', guide =
guide_legend(override.aes = list(alpha = 1))) +
  theme_classic() +
  labs(y = "Weekly Mean Daily Step Count")

#Preparing Data
# Simple linear regression model for ON
ind <- X3$Province == "ON"
```

© 2023 Spilsbury S et al. *JAMA Network Open.*

```
Yon13 <- X3[ind, -c(1, 3, 11, 12, 15 : 17, 19, 20)]

# Include each covariate with low missing as additive effects only
# This means that the whole trend over weeks is adjusted up or down depending on the level of a covariate
lmON13 <- lm(Average.Steps ~ Week * Intervention + gender + age + income_level + current_provider + first_baseline + Experience_mths +
             Engagement_pre + PhysicalActivity_pre, Yon13, na.action = na.exclude)
Yon13$fit3 <- fitted(lmON13)


# Simple linear regression model for BC
ind <- X3$Province == "BC"
Ybc13 <- X3[ind, -c(1, 3, 11, 12, 15 : 17, 19, 20)]

# Same as the Ontario model, where each covariate with low missing is included as an additive effect only
# This means that the whole trend over weeks is adjusted up or down depending on the level of a covariate
lmBC13 <- lm(Average.Steps ~ Week * Intervention + gender + age + income_level + current_provider + first_baseline + Experience_mths +
             Engagement_pre + PhysicalActivity_pre, Ybc13, na.action = na.exclude)
Ybc13$fit3 <- fitted(lmBC13)


# Simple linear regression model for NL
ind <- X3$Province == "NL"
Ynl13 <- X3[ind, -c(1, 3, 11, 12, 15 : 17, 19, 20)]

# Same as the Ontario and BC models, where each covariate with low missing is included as an additive effect only
# This means that the whole trend over weeks is adjusted up or down depending on the level of a covariate
lmNL13 <- lm(Average.Steps ~ Week * Intervention + gender + age + income_level + current_provider + first_baseline + Experience_mths +
             Engagement_pre + PhysicalActivity_pre, Ynl13, na.action = na.exclude)
Ynl13$fit3 <- fitted(lmNL13)

tbl1 <- round(cbind(ON = summary(lmON13)$coefficients[, 1], BC = summary(lmBC13)$coefficients[, 1],
                    NL = summary(lmNL13)$coefficients[, 1]), 1)
pander(tbl1, "Estimates of the simple linear regression model coefficients by Province")

# For each province, use emmeans to get the estimates of interest, i.e. the estimates at Week 18 and Week 13 and use the model coefficients to get the pre and post intervention slopes
```

```
province <- c("ON", "BC", "NL")
preslope <- week13 <- week18 <- diffwk18.13 <- postslope <- vector("numeric", 3)

# Extract Ontario model estimates
preslope[1] <- coef(lmON13)[2]
week13[1] <- summary(emmeans(lmON13, ~ 1, at = list(Week = 13, Intervention =
"pre")))$emmean
week18[1] <- summary(emmeans(lmON13, ~ 1, at = list(Week = 18, Intervention =
"post")))$emmean
diffwk18.13[1] <- week18[1] - week13[1]
postslope[1] <- coef(lmON13)[2] + coef(lmON13)[35]

# Extract BC model estimates
preslope[2] <- coef(lmBC13)[2]
week13[2] <- summary(emmeans(lmBC13, ~ 1, at = list(Week = 13, Intervention =
"pre")))$emmean
week18[2] <- summary(emmeans(lmBC13, ~ 1, at = list(Week = 18, Intervention =
"post")))$emmean
diffwk18.13[2] <- week18[2] - week13[2]
postslope[2] <- coef(lmBC13)[2] + coef(lmBC13)[35]

# Extract NL model estimates
preslope[3] <- coef(lmNL13)[2]
week13[3] <- summary(emmeans(lmNL13, ~ 1, at = list(Week = 13, Intervention =
"pre")))$emmean
week18[3] <- summary(emmeans(lmNL13, ~ 1, at = list(Week = 18, Intervention =
"post")))$emmean
diffwk18.13[3] <- week18[3] - week13[3]
postslope[3] <- coef(lmNL13)[2] + coef(lmNL13)[35]

est1 <- data.frame(province, diffwk18.13, preslope, postslope)
pander(est1, "The difference in the mean Average.Steps between weeks 18 and 13
(diffwdk18.13) and the pre and post intervention rate of change in Average.Steps over weeks
(preslope and postslope respectively) by province")

# Robust covariance for the simple linear regression models
## Inference of the model parameter estimates

# Shown here for the lmNL simple linear regression model that was fit
# Refit the model removing the NA cases for Experience_mths
ind <- !is.na(Ynl13$Experience_mths)
lmNL.4.13 <- lm(Average.Steps ~ Week * Intervention + gender + age + income_level +
current_provider + first_baseline + Experience_mths + Engagement_pre + PhysicalActivity_pre,
droplevels(Ynl13[ind, ]))
V.robust.NL.13 <- vcovCR(lmNL.4.13, cluster = droplevels(Ynl13$user_id[ind]), type = "CR0")
```

© 2023 Spilsbury S et al. *JAMA Network Open.*

```
# Use V.robust.NL to get SEs for the model estimates
# Note, any interactions that include Week with the wk16 or wk17 Intervention levels will be
NA.  These need to be removed from betahat
ind <- is.na(coef(lmNL.4.13))
betahatNL <- coef(lmNL.4.13)[!ind]
seNL <- sqrt(diag(V.robust.NL.13))
z.alpha <- qnorm(0.05 / 2, lower.tail = F)
lowerNL <- betahatNL - (z.alpha * seNL)
upperNL <- betahatNL + (z.alpha * seNL)
zNL <- betahatNL / seNL
pvNL <- 2 * pnorm(q = abs(zNL), lower.tail = FALSE)

tblNL13 <- round(cbind(Estimate = betahatNL, Std.Error = seNL, lower.CI = lowerNL, upper.CI
= upperNL, z.value = zNL,
                 p.value = pvNL), 3)

panderOptions("table.split.table", 90)

pander(tblNL13, "For NL, estimates of the simple linear regression model coefficients, robust
standard errors, lower and upper limits of the 95% confidence interval (CI) for the estimates, and
hypothesis tests that the model coefficients equal zero")

# BC
ind <- !is.na(Ybc13$Experience_mths)
lmBC.4.13 <- lm(Average.Steps ~ Week * Intervention + gender + age + income_level +
current_provider + first_baseline + Experience_mths + Engagement_pre + PhysicalActivity_pre,
droplevels(Ybc13[ind, ]))
V.robust.BC.13 <- vcovCR(lmBC.4.13, cluster = droplevels(Ybc13$user_id[ind]), type =
"CR0")

ind <- is.na(coef(lmBC.4.13))
betahatBC <- coef(lmBC.4.13)[!ind]
seBC <- sqrt(diag(V.robust.BC.13))
lowerBC <- betahatBC - (z.alpha * seBC)
upperBC <- betahatBC + (z.alpha * seBC)
zBC <- betahatBC / seBC
pvBC <- 2 * pnorm(q = abs(zBC), lower.tail = FALSE)

tblBC13 <- round(cbind(Estimate = betahatBC, Std.Error = seBC, lower.CI = lowerBC, upper.CI
= upperBC, z.value = zBC,
                 p.value = pvBC), 4)

pander(tblBC13, "For BC, estimates of the simple linear regression model coefficients, robust
standard errors, lower and upper limits of the 95% confidence interval (CI) for the estimates, and
hypothesis tests that the model coefficients equal zero")
```

© 2023 Spilsbury S et al. *JAMA Network Open.*

```
# ON
ind <- !is.na(Yon13$Experience_mths)
lmON.4.13 <- lm(Average.Steps ~ Week * Intervention + gender + age + income_level +
current_provider + first_baseline + Experience_mths + Engagement_pre + PhysicalActivity_pre,
droplevels(Yon13[ind, ]))
V.robust.ON.13 <- vcovCR(lmON.4.13, cluster = droplevels(Yon13$user_id[ind]), type =
"CR0")

ind <- is.na(coef(lmON.4.13))
betahatON <- coef(lmON.4.13)[!ind]
seON <- sqrt(diag(V.robust.ON.13))
lowerON <- betahatON - (z.alpha * seON)
upperON <- betahatON + (z.alpha * seON)
zON <- betahatON / seON
pvON <- 2 * pnorm(q = abs(zON), lower.tail = FALSE)

tblON13 <- round(cbind(Estimate = betahatON, Std.Error = seON, lower.CI = lowerON,
upper.CI = upperON, z.value = zON,
                   p.value = pvON), 4)

pander(tblON13, "For ON, estimates of the simple linear regression model coefficients, robust
standard errors, lower and upper limits of the 95% confidence interval (CI) for the estimates, and
hypothesis tests that the model coefficients equal zero")

## Inference of the post-intervention rate of change
# A linear combination of the model coefficients is needed to get the post-intervention rate of
change and its standard error.  And the vector L is used to specify the parameters needed from
betahat to get the appropriate combination.  Note that this means that L must be the same length
as betahat.  Only for those models that have a two-way interaction between Week and
Intervention and all covariates included as additive effects only (i.e. no 3-way interactions), the
contrast L must include 1's at the indices in betahat for Week and Week:Interventionpost.

# NL
L <- rep(0, length(betahatNL))
ind <- names(betahatNL) %in% c("Week")
L[ind] <- 1

estimate <- L %*% betahatNL
SE <- sqrt(t(L) %*% V.robust.NL.13 %*% L)
lower <- estimate - (z.alpha * SE)
upper <- estimate + (z.alpha * SE)
z <- estimate / SE
pv <- 2 * pnorm(q = abs(z), lower.tail = FALSE)
preNL13 <- round(c(Estimate = estimate, Std.Error = SE, lower.CI = lower, upper.CI = upper,
z.value = z, p.value = pv), 3)
```

© 2023 Spilsbury S et al. *JAMA Network Open.*

```
# BC
L <- rep(0, length(betahatBC))
ind <- names(betahatBC) %in% c("Week")
L[ind] <- 1
estimate <- L %*% betahatBC
SE <- sqrt(t(L) %*% V.robust.BC.13 %*% L)
lower <- estimate - (z.alpha * SE)
upper <- estimate + (z.alpha * SE)
z <- estimate / SE
pv <- 2 * pnorm(q = abs(z), lower.tail = FALSE)
preBC13 <- round(c(Estimate = estimate, Std.Error = SE, lower.CI = lower, upper.CI = upper,
z.value = z, p.value = pv), 3)

#ON
L <- rep(0, length(betahatON))
ind <- names(betahatON) %in% c("Week")
L[ind] <- 1
estimate <- L %*% betahatON
SE <- sqrt(t(L) %*% V.robust.ON.13 %*% L)
lower <- estimate - (z.alpha * SE)
upper <- estimate + (z.alpha * SE)
z <- estimate / SE
pv <- 2 * pnorm(q = abs(z), lower.tail = FALSE)
preON13 <- round(c(Estimate = estimate, Std.Error = SE, lower.CI = lower, upper.CI = upper,
z.value = z, p.value = pv), 3)

pander(rbind(preNL13, preBC13, preON13), "95% CI and hypothesis test for the post-
intervention rate of change in average steps for NL, BC, and ON")

# NL
L <- rep(0, length(betahatNL))
ind <- names(betahatNL) %in% c("Week", "Week:Interventionpost")
L[ind] <- 1

estimate <- L %*% betahatNL
SE <- sqrt(t(L) %*% V.robust.NL.13 %*% L)
lower <- estimate - (z.alpha * SE)
upper <- estimate + (z.alpha * SE)
z <- estimate / SE
pv <- 2 * pnorm(q = abs(z), lower.tail = FALSE)
postNL13 <- round(c(Estimate = estimate, Std.Error = SE, lower.CI = lower, upper.CI = upper,
z.value = z, p.value = pv), 3)

# BC
L <- rep(0, length(betahatBC))
ind <- names(betahatBC) %in% c("Week", "Week:Interventionpost")
```

© 2023 Spilsbury S et al. *JAMA Network Open.*

```
L[ind] <- 1
estimate <- L %*% betahatBC
SE <- sqrt(t(L) %*% V.robust.BC.13 %*% L)
lower <- estimate - (z.alpha * SE)
upper <- estimate + (z.alpha * SE)
z <- estimate / SE
pv <- 2 * pnorm(q = abs(z), lower.tail = FALSE)
postBC13 <- round(c(Estimate = estimate, Std.Error = SE, lower.CI = lower, upper.CI = upper,
z.value = z, p.value = pv), 3)

#ON
L <- rep(0, length(betahatON))
ind <- names(betahatON) %in% c("Week", "Week:Interventionpost")
L[ind] <- 1
estimate <- L %*% betahatON
SE <- sqrt(t(L) %*% V.robust.ON.13 %*% L)
lower <- estimate - (z.alpha * SE)
upper <- estimate + (z.alpha * SE)
z <- estimate / SE
pv <- 2 * pnorm(q = abs(z), lower.tail = FALSE)
postON13 <- round(c(Estimate = estimate, Std.Error = SE, lower.CI = lower, upper.CI = upper,
z.value = z, p.value = pv), 3)

pander(rbind(postNL13, postBC13, postON13), "95% CI and hypothesis test for the post-
intervention rate of change in average steps for NL, BC, and ON")

## Comparing estimated slopes
estimate <- postBC13[1] - postNL13[1]
SEpooled <- sqrt((postBC13[2] ^ 2) + (postNL13[2] ^ 2))
lower <- estimate - (z.alpha * SEpooled)
upper <- estimate + (z.alpha * SEpooled)
z <- estimate / SEpooled
pv <- 2 * pnorm(q = abs(z), lower.tail = FALSE)

tbl13 <- matrix(round(c(estimate, SEpooled, lower, upper, z, pv), 3), 1, 6)
colnames(tbl13) <- c("Difference", names(postBC13)[2 : 6])

estimate <- postON13[1] - postBC13[1]
SEpooled <- sqrt((postON13[2] ^ 2) + (postBC13[2] ^ 2))
lower <- estimate - (z.alpha * SEpooled)
upper <- estimate + (z.alpha * SEpooled)
z <- estimate / SEpooled
pv <- 2 * pnorm(q = abs(z), lower.tail = FALSE)

tbl13 <- matrix(round(c(estimate, SEpooled, lower, upper, z, pv), 3), 1, 6)
colnames(tbl13) <- c("Difference", names(postON13)[2 : 6])
```

© 2023 Spilsbury S et al. *JAMA Network Open.*

```
estimate <- postON13[1] - postNL13[1]
SEpooled <- sqrt((postON13[2] ^ 2) + (postNL13[2] ^ 2))
lower <- estimate - (z.alpha * SEpooled)
upper <- estimate + (z.alpha * SEpooled)
z <- estimate / SEpooled
pv <- 2 * pnorm(q = abs(z), lower.tail = FALSE)

tbl13 <- matrix(round(c(estimate, SEpooled, lower, upper, z, pv), 3), 1, 6)
colnames(tbl13) <- c("Difference", names(postON13)[2 : 6])

#Intercept
#Within
#Pre
#NL
LIntPre <- matrix(c(1, 12, 0, 0, 0, 0, 0, 0, 0.272, .009, 35.78, .137, .09, .118, .138, .120, .110, .01,
.029, .161, .048, .002, .007, .017, .66, 5307, 17.93, 9.38, 5863, 0), nrow = 1)

estimate <- LIntPre %*% betahatNL
SE <- sqrt(LIntPre %*% V.robust.NL.13 %*% t(LIntPre))
lower <- estimate - (z.alpha * SE)
upper <- estimate + (z.alpha * SE)
z <- estimate / SE
pv <- 2 * pnorm(q = abs(z), lower.tail = FALSE)
preIntNL13 <- round(c(Estimate = estimate, Std.Error = SE, lower.CI = lower, upper.CI = upper,
z.value = z, p.value = pv), 3)

#BC
LIntPre <- matrix(c(1, 12, 0, 0, 0, 0, 0, 0, 0.312, .02, 36.51, .121, .07, .125, .164, .135, .106, .007,
.031, .159, .034, .103, .063, .022, .597, 5883, 17.54, 10.11, 6712, 0), nrow = 1)

estimate <- LIntPre %*% betahatBC
SE <- sqrt(LIntPre %*% V.robust.BC.13 %*% t(LIntPre))
lower <- estimate - (z.alpha * SE)
upper <- estimate + (z.alpha * SE)
z <- estimate / SE
pv <- 2 * pnorm(q = abs(z), lower.tail = FALSE)
preIntBC13 <- round(c(Estimate = estimate, Std.Error = SE, lower.CI = lower, upper.CI = upper,
z.value = z, p.value = pv), 3)

#ON
LIntPre <- matrix(c(1, 12, 0, 0, 0, 0, 0, 0, 0.339, .015, 33.92, .114, .083, .124, .151, .129, .105,
.012, .04, .148, .045, .01, .109, .021, .676, 5751, 12.75, 9.37, 6431, 0), nrow = 1)

estimate <- LIntPre %*% betahatON
SE <- sqrt(LIntPre %*% V.robust.ON.13 %*% t(LIntPre))
```

© 2023 Spilsbury S et al. *JAMA Network Open.*

```
lower <- estimate - (z.alpha * SE)
upper <- estimate + (z.alpha * SE)
z <- estimate / SE
pv <- 2 * pnorm(q = abs(z), lower.tail = FALSE)
preIntON13 <- round(c(Estimate = estimate, Std.Error = SE, lower.CI = lower, upper.CI = upper,
z.value = z, p.value = pv), 3)

PreInt <- rbind(preIntNL13, preIntBC13, preIntON13)

#Post
LIntPost <- matrix(c(1, 21, 0, 0, 0, 0, 0, 1, 0.272, .009, 35.78, .137, .09, .118, .138, .120, .110,
.01, .029, .161, .048, .002, .007, .017, .66, 5307, 17.93, 9.38, 5863, 21), nrow = 1)

estimate <- LIntPost %*% betahatNL
SE <- sqrt(LIntPost %*% V.robust.NL.13 %*% t(LIntPost))
lower <- estimate - (z.alpha * SE)
upper <- estimate + (z.alpha * SE)
z <- estimate / SE
pv <- 2 * pnorm(q = abs(z), lower.tail = FALSE)
postIntNL13 <- round(c(Estimate = estimate, Std.Error = SE, lower.CI = lower, upper.CI =
upper, z.value = z, p.value = pv), 3)

#BC
LIntPost <- matrix(c(1, 21, 0, 0, 0, 0, 0, 1, 0.312, .02, 36.51, .121, .07, .125, .164, .135, .106,
.007, .031, .159, .034, .103, .063, .022, .597, 5883, 17.54, 10.11, 6712, 21), nrow = 1)

estimate <- LIntPost %*% betahatBC
SE <- sqrt(LIntPost %*% V.robust.BC.13 %*% t(LIntPost))
lower <- estimate - (z.alpha * SE)
upper <- estimate + (z.alpha * SE)
z <- estimate / SE
pv <- 2 * pnorm(q = abs(z), lower.tail = FALSE)
postIntBC13 <- round(c(Estimate = estimate, Std.Error = SE, lower.CI = lower, upper.CI =
upper, z.value = z, p.value = pv), 3)

#ON
LIntPost <- matrix(c(1, 21, 0, 0, 0, 0, 0, 1, 0.339, .015, 33.92, .114, .083, .124, .151, .129, .105,
.012, .04, .148, .045, .01, .109, .021, .676, 5751, 12.75, 9.37, 6431, 21), nrow = 1)

estimate <- LIntPost %*% betahatON
SE <- sqrt(LIntPost %*% V.robust.ON.13 %*% t(LIntPost))
lower <- estimate - (z.alpha * SE)
upper <- estimate + (z.alpha * SE)
z <- estimate / SE
pv <- 2 * pnorm(q = abs(z), lower.tail = FALSE)
```

© 2023 Spilsbury S et al. *JAMA Network Open.*

```
postIntON13 <- round(c(Estimate = estimate, Std.Error = SE, lower.CI = lower, upper.CI =
upper, z.value = z, p.value = pv), 3)

PostInt <- rbind(postIntNL13, postIntBC13, postIntON13)

#Between
#Pre
estimate <- preIntON13[1] - preIntBC13[1]
SEpooled <- sqrt((preIntON13[2] ^ 2) + (preIntBC13[2] ^ 2))
lower <- estimate - (z.alpha * SEpooled)
upper <- estimate + (z.alpha * SEpooled)
z <- estimate / SEpooled
pv <- 2 * pnorm(q = abs(z), lower.tail = FALSE)

tblpreIntONBC13 <- matrix(round(c(estimate, SEpooled, lower, upper, z, pv), 3), 1, 6)
colnames(tblpreIntONBC13) <- c("Difference", names(preIntON13)[2 : 6])

#ON-NL
estimate <- preIntON13[1] - preIntNL13[1]
SEpooled <- sqrt((preIntON13[2] ^ 2) + (preIntNL13[2] ^ 2))
lower <- estimate - (z.alpha * SEpooled)
upper <- estimate + (z.alpha * SEpooled)
z <- estimate / SEpooled
pv <- 2 * pnorm(q = abs(z), lower.tail = FALSE)

tblpreIntONNL13 <- matrix(round(c(estimate, SEpooled, lower, upper, z, pv), 3), 1, 6)
colnames(tblpreIntONNL13) <- c("Difference", names(preIntON13)[2 : 6])

#BC-NL
estimate <- preIntBC13[1] - preIntNL13[1]
SEpooled <- sqrt((preIntBC13[2] ^ 2) + (preIntNL13[2] ^ 2))
lower <- estimate - (z.alpha * SEpooled)
upper <- estimate + (z.alpha * SEpooled)
z <- estimate / SEpooled
pv <- 2 * pnorm(q = abs(z), lower.tail = FALSE)

tblpreIntBCNL13 <- matrix(round(c(estimate, SEpooled, lower, upper, z, pv), 3), 1, 6)
colnames(tblpreIntBCNL13) <- c("Difference", names(preIntBC13)[2 : 6])

#Post
#ON-BC
estimate <- postIntON13[1] - postIntBC13[1]
SEpooled <- sqrt((postIntON13[2] ^ 2) + (postIntBC13[2] ^ 2))
lower <- estimate - (z.alpha * SEpooled)
upper <- estimate + (z.alpha * SEpooled)
z <- estimate / SEpooled
```

© 2023 Spilsbury S et al. *JAMA Network Open.*

```
pv <- 2 * pnorm(q = abs(z), lower.tail = FALSE)

tblpostIntONBC13 <- matrix(round(c(estimate, SEpooled, lower, upper, z, pv), 3), 1, 6)
colnames(tblpostIntONBC13) <- c("Difference", names(postIntON13)[2 : 6])

#ON-NL
estimate <- postIntON13[1] - postIntNL13[1]
SEpooled <- sqrt((postIntON13[2] ^ 2) + (postIntNL13[2] ^ 2))
lower <- estimate - (z.alpha * SEpooled)
upper <- estimate + (z.alpha * SEpooled)
z <- estimate / SEpooled
pv <- 2 * pnorm(q = abs(z), lower.tail = FALSE)

tblpostIntONNL13 <- matrix(round(c(estimate, SEpooled, lower, upper, z, pv), 3), 1, 6)
colnames(tblpostIntONNL13) <- c("Difference", names(postIntON13)[2 : 6])

#BC-NL
estimate <- postIntBC13[1] - postIntNL13[1]
SEpooled <- sqrt((postIntBC13[2] ^ 2) + (postIntNL13[2] ^ 2))
lower <- estimate - (z.alpha * SEpooled)
upper <- estimate + (z.alpha * SEpooled)
z <- estimate / SEpooled
pv <- 2 * pnorm(q = abs(z), lower.tail = FALSE)

tblpostIntBCNL13 <- matrix(round(c(estimate, SEpooled, lower, upper, z, pv), 3), 1, 6)
colnames(tblpostIntBCNL13) <- c("Difference", names(postIntBC13)[2 : 6])
```

© 2023 Spilsbury S et al. *JAMA Network Open.*
