# Supplementary material for: Reduction of Financial Health Incentives and Changes in Physical Activity
Source: JAMA Netw Open. 2023 Nov 8;6(11):e2342663. doi: 10.1001/jamanetworkopen.2023.42663 (PMC10632955; doi:10.1001/jamanetworkopen.2023.42663)
Supplement: Supplement 2. — Data Sharing Statement [file jamanetwopen-e2342663-s002.pdf]

Data Sharing Statement

Spilsbury. Reduction of Financial Health Incentives and Changes in Physical Activity. *JAMA Netw Open*. Published November 08, 2023. doi:10.1001/jamanetworkopen.2023.42663

Data
Data available: Yes
Data types: Deidentified participant data
How to access data: The datasets generated during and/or analysed during the current study are not publicly available due to a lack of authorization to share individual-level data, but de-identified participant data are available from the corresponding author (marc.mitchell@uwo.ca) on reasonable request and with appropriate reason when accompanied by study protocol and analysis plan. Data will be shared after the approval of a proposal by a committee of the current research team with a signed data access agreement.
When available: With publication

Supporting Documents
Document types: Statistical/analytic code
How to access documents: The code for our primary data analysis is provided in Supplement 12.
When available: beginning date: 07-11-2023

Additional Information
Who can access the data: JAMA Network Open editors and reviewers, as well as others reading the published paper.
Types of analyses: For the primary analysis.
Mechanisms of data availability: With investigator support after approval of a proposal and with a signed data access agreement.
